# Inhibition of Gasdermin D-Mediated Pyroptosis Attenuates the Severity of Seizures and Astroglial Damage in Kainic Acid-Induced Epileptic Mice

**DOI:** 10.3389/fphar.2021.751644

**Published:** 2022-01-28

**Authors:** Lu Xia, Lu Liu, Yiying Cai, Yiying Zhang, Fangchao Tong, Qiang Wang, Jing Ding, Xin Wang

**Affiliations:** ^1^ Department of Neurology, Zhongshan Hospital, Fudan University, Shanghai, China; ^2^ Department of the State Key Laboratory of Medical Neurobiology, The institutes of Brain Science and the Collaborative Innovation Center for Brain Science, Fudan University, Shanghai, China

**Keywords:** epilepsy, GSDMD, pyroptosis, astrocyte, inflammation

## Abstract

**Objective:** Our study aimed to explore whether gasdermin D (GSDMD)-mediated pyroptosis is involved in the mechanism of kainic acid-induced seizures.

**Methods:** C57BL/6 mice were randomly divided into sham and epilepsy groups. The epilepsy group was intrahippocampally injected with kainic acid to induce status epilepticus (SE), and the sham group was injected with an equal volume of saline. Dimethyl fumarate (DMF) was used as the GSDMD N-terminal fragments (GSDMD-N) inhibitor and suspended in 0.5% sodium carboxymethyl cellulose (CMC) for orally administration. The epilepsy group was divided into SE + CMC and SE + DMF groups. In the SE + DMF group, DMF was orally administered for 1 week before SE induction and was continued until the end of the experiment. An equal volume of CMC was administered to the sham and SE + CMC groups. Recurrent spontaneous seizures (SRSs) were monitored for 21 days after SE. Western blot analysis and immunofluorescent staining was performed.

**Results:** The expression of GSDMD increased at 7–21 days post-SE, and GSDMD-N expression was significantly elevated 7 days after SE in both ipsilateral and contralateral hippocampus. GSDMD-positive cells were co-labeled with astrocytes, but not neurons or microglia. Astroglial damage occurs following status epilepticus (SE). Damaged astrocytes showed typical clasmatodendrosis in the CA1 region containing strong GSDMD expression at 7–21 days post-SE, accompanied by activated microglia. In the SE + DMF group, the expression of GSDMD-N was significantly inhibited compared to that in the SE + CMC group. After administration of DMF, SRSs at 7–21 days after SE were significantly decreased, and the number of clasmatodendritic astrocytes, microglia, and the expression of inflammatory factors such as IL-1β and IL-18 were significantly attenuated.

**Conclusion:** GSDMD-mediated pyroptosis is involved in the mechanism of kainic acid-induced seizures. Our study provides a new potential therapeutic target for seizure control.

## Introduction

Epilepsy is a common neurological disease that affects over 70 million people worldwide, with an annual incidence of approximately 50–100 per 100,000 persons (Thijs et al., 2019). The underlying mechanisms of epilepsy are not yet fully understood. Accumulating evidence indicates that neuroinflammation plays an important role in the severity of epilepsy (Aronica et al., 2017). Previous studies have demonstrated that neuroinflammation can promote neuronal hyperexcitability and contributes to seizures, neuronal cell loss, and maladaptive synaptic plasticity (Vezzani et al., 2019). Substantial changes in neuroglial cells were found in both epileptic animal models and resected brain tissues from epileptic patients who underwent surgery, which are important factors in the development of neuroinflammation (Rana and Musto, 2018; Feng et al., 2019; Sanz and Garcia-Gimeno, 2020).

Pyroptosis is a new form of inflammatory programmed cell death that is characterized by cell swelling, cell membrane disruption, the release of cellular contents, and inflammatory factors (Shi et al., 2017). Pyroptosis occurs through two pathways: the canonical and non-canonical pathways. In the canonical pathway, in response to extracellular or intracellular danger signals, the nucleotide oligomerization domain (NOD)-like receptor (NLR) family pyrin domain-containing 1 and 3 (NLRP1 and NLRP3), or the absent in melanoma 2 (AIM2) protein, recruit pro-caspase-1 to form canonical inflammasomes and activate caspase-1. Activated caspase-1 cleaves gasdermin D (GSDMD) to release the N-terminal fragment (GSDMD-N), which forms pores in the cell membrane, leading to the release of inflammatory factors and damage-associated molecular patterns (DAMPs), such as high-mobility group box 1 (HMGB1) and adenosine 5′-triphosphate (ATP), resulting in cell death (Kayagaki et al., 2015; Shi et al., 2015). In particular, mature interleukin (IL)-1 family proteins, such as cytokines IL-1β and IL-18, were preferentially released from the GSDMD pore by electrostatic filtering (Xia et al., 2021). In the non-canonical pathway, GSDMD is cleaved by caspases-4/5/11 to induce pyroptosis (Huang et al., 2019). It has been found that in patients with mesial temporal lobe epilepsy, NLRP3 and NLRP1 inflammasomes are upregulated, which may be responsible for the increased hippocampal expression of caspase-1 and IL-1β (Cristina de Brito Toscano et al., 2021). GSDMD cleavage serves as an important checkpoint in pyroptosis in both canonical and non-canonical pathways and plays an important role in central nervous system diseases such as Alzheimer’s disease, cerebral ischemia, and multiple sclerosis (McKenzie et al., 2018; Han et al., 2020; Li et al., 2020). However, the expression levels and potential role of GSDMD in seizures remain unknown.

Astrocytes are the most abundant glial cell type in the nervous system and are essential for maintaining brain homeostasis. Previous studies have demonstrated that lipopolysaccharide (LPS) exposure stimulates the upregulation of GSDMD and caspase-1 protein in cultured astrocytes, indicating that LPS triggered astrocyte pyroptosis (Sun et al., 2019). The upregulation of GSDMD expression in astrocytes has also been confirmed in hypoxic animal models (Jiang et al., 2021). Although the expression of GSDMD in astrocytes post seizures is unclear, it has been found that HMGB1 staining in the nuclear and perinuclear region was increased in astrocytes after seizures (Maroso et al., 2010). Administration of a caspase-1 inhibitor (VX-765) significantly reduced chronic epileptic activity, and the drug effect was associated with the inhibition of IL-1β synthesis in astrocytes (Maroso et al., 2011). Moreover, it has been reported that astroglial damage occurs in the CA1 region of hippocampus following status epilepticus (SE), which is termed clasmatodendrosis (Penfield, 1928). This was considered as lysosome-derived autophagic death because of immunoreactivity with autophagic markers, such as lysosome-associated membrane protein 1 and lipidated LC3 (Ryu et al., 2011; Hase et al., 2018). Furthermore, it has been reported that astroglial damage induced by SE correlate with altered electrophysiological properties (Kim et al., 2008). Clasmatodendrotic astrocytes are characterized by extensive swelling and vacuolization of cell bodies, disintegrated/beaded processes, and nuclear dissolution, which is partly similar to pyroptosis and is therefore difficult to distinguish based on the morphological characteristic of tissues. Thus, the aim of the present study was to explorewhether GSDMD-mediated pyroptosis is associated with astroglial damage (clasmatodendrosis) induced by seizures and is involved in the mechanism of seizures.

The aim of the present study was to explore whether GSDMD-mediated pyroptosis is involved in the mechanism of kainic acid-induced seizures and whether inhibiting GSDMD-N can attenuate pyroptosis and the severity of seizures.

## Methods and Materials

### Animals

Male adult C57BL/6 mice (22–26 g) aged 8–10 weeks were housed in cages at an ambient temperature (22–25°C) and maintained under a standard 12/12 h light/dark cycle. The experiment was done in accordance with the guidelines of the National Institutes of Health. All experiments have been approved by the Ethics Committee of Zhongshan Hospital of Fudan University (Approval number 2019-151, Shanghai, China). All measures were taken to minimise animal suffering and to reduce the number of animals used.

### Establishment of a Kainic Acid-Induced Epileptic Mouse Model

The epileptic model induced by intrahippocampal injection of kainic acid in mice has pathological characteristics similar to those of patients with temporal lobe epilepsy. During surgery, mice were anesthetized with an intraperitoneal injection of 0.01 ml/g 1% pentobarbital sodium solution. For intrahippocampal injections, a 2 μl microsyringe was stereotaxically implanted into the hippocampal area at the following coordinates: anteroposterior (AP) = −2.7 mm, mediolateral (ML) = −1.8 mm, and dorsoventral (DV) = −1.7 mm. Kainic acid (0.4 µg in 0.8 μl saline; Sigma, United States) was injected over a duration of 2 min. The needle was maintained *in situ* for an additional 5 min to avoid reflux along the injection track. Sham surgery mice were injected with 0.8 μl of saline. The Racine scale was used to evaluate seizure severity (Racine, 1972); mice that presented with seizures greater than or equal to Racine stage 4 were considered positive for successful SE. These mice were included in subsequent studies.

### Grouping and Treatment

Dimethyl fumarate (DMF) can react with GSDMD at critical cysteine residues to form S (2-succinyl)-cysteine. GSDMD succination prevents its interaction with caspases, limiting its progression and oligomerization to form GSDMD-N (Humphries et al., 2020). DMF, as a GSDMD-N inhibitor, was purchased from Sigma-Aldrich (St. Louis, MO, United States, Cat No.242926) and suspended in 0.5% sodium carboxymethyl cellulose (CMC) (Selleck, United States, Cat No.S6703). DMF (100 mg/kg/d) at a volume of 0.2 ml was administered by gastric gavage. Mice were divided into the sham and SE groups. The SE mice were divided into two subgroups: 1) the SE + DMF group, which was administered 100 mg/kg/d DMF for 1 week before SE induction and was continued until the end of the experiment; and 2) the SE + CMC group, which was given an equal volume of vehicle (0.5% CMC) instead of DMF. The sham group was administered CMC, as described for the SE + CMC group.

### Protein Extraction and Western Blot Analysis

The mice were deeply anesthetized with 4% chloral hydrate and then euthanized by cervical dislocation. The hippocampal tissues were carefully dissected from the brain, total protein was extracted using a tissue protein extraction reagent (Beyotime Institute of Biotechnology, China), and the total protein concentration was determined using a bicinchoninic acid (BCA) protein assay kit (Beyotime, China). The protein extract (20 µg) was electrophoresed on a 12.5% sodium dodecyl sulfate polyacrylamide gel (SDS-PAGE) and then transferred onto 0.45 µm nitrocellulose membranes (Merck Millipore Ltd., Ireland). The membranes were blocked with 5% nonfat milk at room temperature for 1 h and were then incubated overnight at 4°C with primary antibodies, including rabbit anti-GSDMD (1:1000, Abcam ab219800), rabbit anti-GSDMD-N (1:1000, Abcam ab219800), mouse anti-pro-IL-1β (1:1000, Cell Signaling Technology, United States, cat #12242), rabbit anti-cleaved-IL-1β (1:500, Cell Signaling Technology, United States, cat #63124), rabbit anti-pro-caspase-1 (1:1000, Cell Signaling Technology, cat #24232) and rabbit anti-cleaved-caspase-1 (1:500, Cell Signaling Technology, cat #89332). Mouse anti-β-actin antibody (1:1000, Cell Signaling Technology, cat #3700) was used as an internal reference. The membranes were then incubated with goat anti-rabbit IgG secondary antibody (1:1000, Cell Signaling Technology, cat #7074) and horse anti-mouse IgG secondary antibody (1:1000, Cell Signaling Technology, cat #7076) for 1 h at room temperature. The images of the bands were captured using Tanon Image software (version 4100, Shanghai, China). The optical density (OD) value was normalized to that of β-actin.

### Immunofluorescent Staining

The mice were deeply anesthetized with 4% chloral hydrate, and after perfusing with 4°C saline and 4% paraformaldehyde in phosphate-buffered saline (PBS), the mice were decapitated and the brain was quickly removed from the skull. The brain was stored in 4% paraformaldehyde solution at 4°C for 24 h and then moved to 10% sucrose in 0.1 m PBS at 4°C until it sank. It was then transferred to 20% sucrose solution and finally moved to a 30% sucrose solution at 4°C until it sank.

Coronal sections (20 µm) were sliced from the dorsal side of the hippocampus using a frozen slicer (CM 1950, Leica, Heidelberg, Germany). The sections were washed 3 times with PBS for 5 min each time, blocked with immunol staining blocking buffer (Beyotime Institute of Biotechnology, China) for 30 min, and were then incubated with the primary antibody overnight at 4°C. The sections were washed 3 times with PBS for 5 min each time, incubated with secondary antibodies for 1 h at room temperature, and mounted with antifade medium containing 4′,6-diamidino-2-phenylindole (DAPI). The primary antibodies used in this study were as follows: GSDMD (1:200, Affinity, cat #AF4012), glial fibrillary acidic protein (GFAP) (1:1000, Millipore, cat #MAB360), ionized calcium binding adaptor molecule 1 (Iba1) (1:500, Novus Biologicals, cat #NB100-1028), and neuronal nuclear protein (NeuN) (1:500, Millipore, cat #MAB377). The secondary antibodies (1:1000) and Alexa Fluor@488- and 555-conjugated antibodies were purchased from Abcam (Cambridge, United Kingdom). Images of the sections were captured using a fluorescence microscope (Olympus/BX51, Japan). We selected CA1, CA3, and DG subfields of the hippocampus from 2 to 3 coronal slices of each mouse for analysis. For each section, a region of interest (ROI) of 460 × 345 µm (158,700 µm^2^) was employed to count the number of labeled cells at ×20 magnification.

### Enzyme-Linked Immunosorbent Assay

Cytokines (IL-1β, and IL-18) in the ipsilateral hippocampus were measured using a Ray Biotech kit (America). Briefly, adding 100 μl of standard or sample to each well and incubate for 150 min. Next, adding 300 μl washing buffer to each well and wash 3 times. Adding 100 μl of diluted Streptavidin-HRP to each well and incubate for 45 min. After washing 3 times, adding 100 μl Substrate Solation to each well, and incubate in the dark at room temperature for about 30 min. Finally, adding 50 µl stop solution to each well, and testing the results within 20 min.

### Statistical Methods

GraphPad Prism 8 software was used for the statistical analyses. Mean ± standard error of the mean (SEM) was used to represent the data. Two independent samples were statistically analyzed by Student’s t-test, and two-sided *p* < 0.05 was considered statistically significant.

## Results

### GSDMD and Pyroptosis-Related Molecules are Expressed in Kainic Acid-Induced Epileptic Mice

**FIGURE 1:**
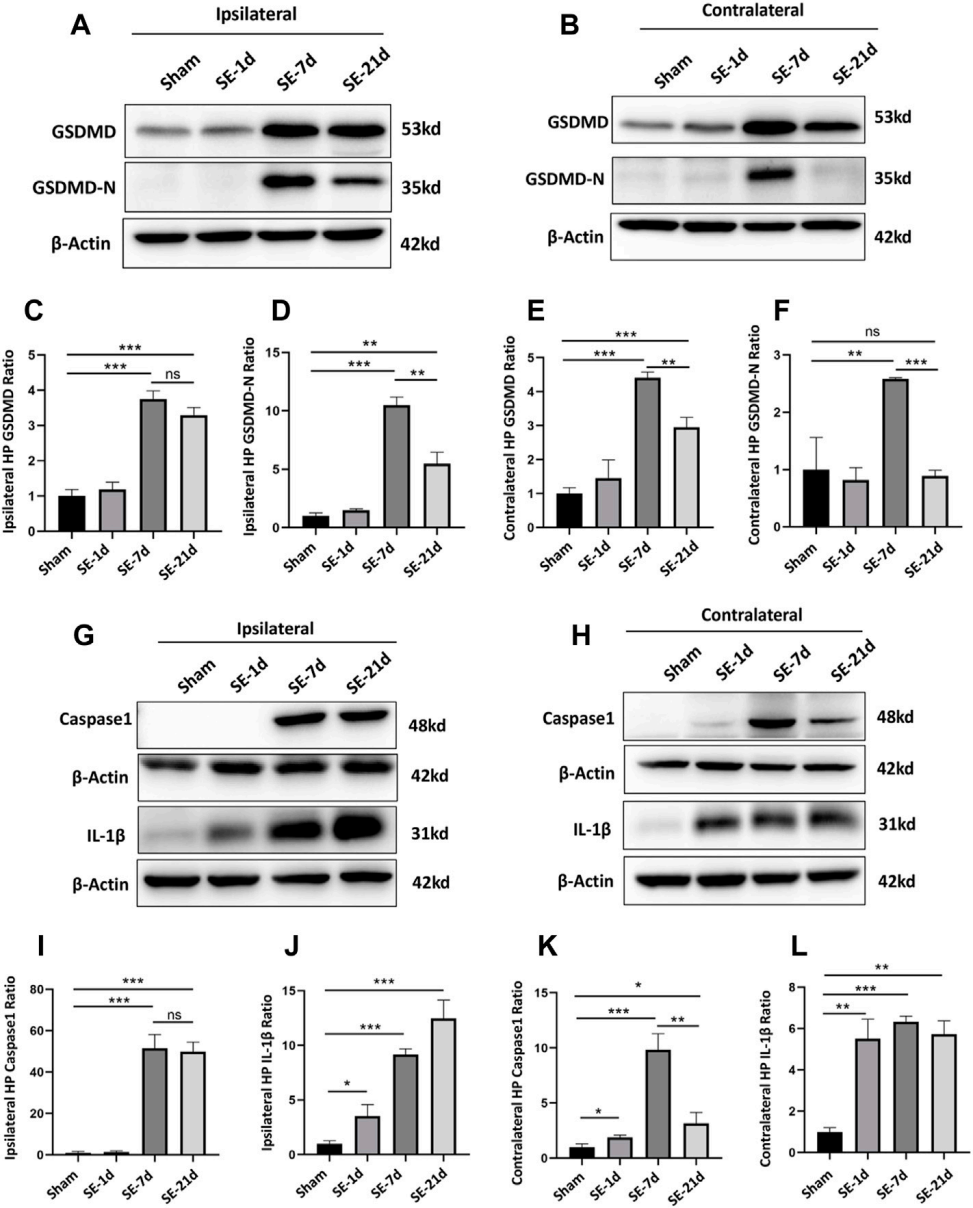
Expression of GSDMD and pyroptosis-related molecules was significantly increased after kainic acid-induced SE. **(A, B)** WB bands of GSDMD, GSDMD-N and β-actin proteins in the ipsilateral and contralateral hippocampus. **(C–F)** Statistical analyses of GSDMD, GSDMD-N and β-actin proteins in the ipsilateral and contralateral hippocampus. **(G, H)** WB bands of caspase-1, IL-1β, and β-actin proteins in the ipsilateral and contralateral hippocampus. **(I–L)** Statistical analyses of caspase-1, IL-1β, and β-actin proteins in the ipsilateral and contralateral hippocampus (n = 3 in each group, *p < 0.05, **p < 0.01, and ***p < 0.001).

GSDMD is a key molecular executor of pyroptosis. We used western blotting (WB) to quantify the expression of GSDMD in the ipsilateral and contralateral hippocampus on days 1, 7, and 21 after kainic acid-induced SE, relative to that in the sham group. The protein expression of full-length GSDMD increased significantly at 7–21 days post-SE in both the ipsilateral and contralateral hippocampus. Moreover, the expression level of the pore-forming N-terminal GSDMD was significantly higher than that of other groups at 7 days post-SE in both the ipsilateral and contralateral hippocampus (Figures 1A–F). The levels of caspase-1 and IL-1β, the other two important molecules of the pyroptosis pathway, also increased significantly in both the ipsilateral and contralateral hippocampus after SE (Figures 1G–L).

### Damaged Astrocytes (Clasmatodendrosis) Were Detected in the Hippocampal CA1 Area and Co-Labeled With GSDMD

**FIGURE 2:**
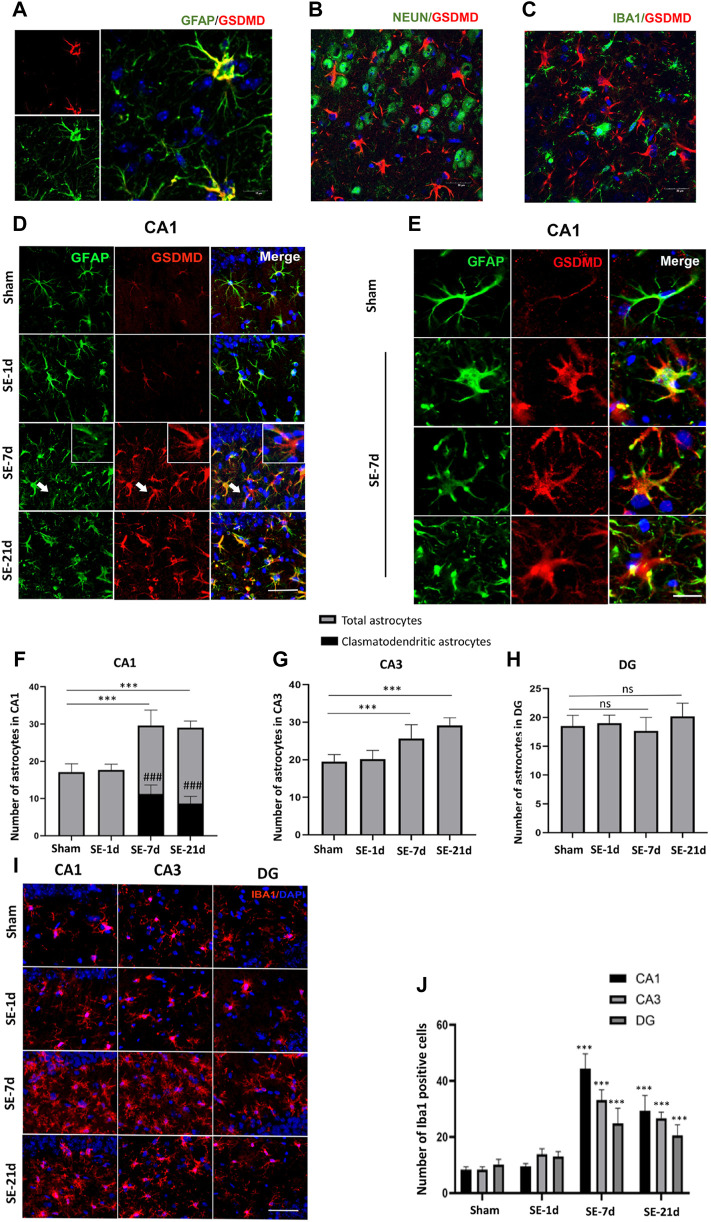
Clasmatodendritic astrocytes co-labeled with GSDMD after SE. **(A–C)** Representative images of GSDMD (red)/GFAP (green), GSDMD (red)/NeuN (green), and GSDMD (red)/Iba-1 (green) staining in hippocampal slices from mice at day 7 after kainic acid injection. **(D)** Microphotographs of GSDMD (red) and GFAP (green) staining in the CA1 region of the hippocampus in the sham, SE-1d, SE-7d, and SE-21d groups (bar = 50 µm). **(E)** Typical GSDMD-positive clasmatodendritic astrocytes in the CA1 region of the hippocampus at 7 days after SE (bar = 12.5 µm). **(F–H)** Statistical analyses of the number of GSDMD-positive clasmatodendritic astrocytes and total astrocytes in the CA1, CA3, and DG regions (n = 3 in each group, asterisks represents the total astrocytes in comparison with the sham group, *p < 0.05, **p < 0.01, and ***p < 0.001; well number represents the clasmatodendritic astrocytes in comparison with the sham group, ###*p* < 0.001). **(I)** Microphotographs of Iba1 (red) staining in the CA1, CA3, and DG regions of the hippocampus in the sham, SE-1d, SE-7d, and SE-21d groups. **(J)** Statistical analyses of the number of Iba1-positive cells in the CA1, CA3, and DG regions (n = 3 in each group, asterisks represent the comparison with the sham group, *p < 0.05, **p < 0.01 and ***p < 0.001).

Immunofluorescence results showed distinct glial fibrillary acidic protein (GFAP) loss in the ipsilateral CA1 and CA3 regions of the hippocampus. The closer the Kainic acid injected , the greater the GFAP lost (Supplementary Figure S1). To eliminate the interference of modeling operations and modeling drugs, immunofluorescence analysis was performed on the contralateral hippocampus; to determine the cell type that underwent pyroptosis, immunofluorescence co-labeling was performed. GFAP, Iba1, and NeuN are common markers for astrocytes, microglia, and neurons, respectively. The results showed that GSDMD-positive cells were co-labeled with GFAP-immunoreactive cells (astrocytes) but not with NeuN-positive (neurons) or Iba1-positive (microglia) cells (Figures 2A–C). Seven days after SE, astrocytes showed typical clasmatodendrosis in the CA1 region, which was characterized by extensive swelling and vacuolization of cell bodies, disintegrated/beaded processes, and strong GSDMD expression (Figures 2D–E). Consistent with previous studies, clasmatodendritic astrocytes were only observed in the CA1 region. The results of the statistical analyses showed that the total number of astrocytes increased significantly in the CA1 and CA3 regions of the hippocampus at 7–21 days post-SE, while no significant differences were observed in the DG area (Figures 2F–H). Meanwhile, we found that astrocytes without clasmatodendrosis in the CA3 and DG regions of the hippocampus were still co-labeled with GSDMD (Supplementary Figure S2). Microglia, as innate immune cells in the central nervous system, proliferate and activate rapidly once stimulated by “risk factors.” We used Iba1-labeled microglia to analyze whether the release of cellular contents by astrocyte pyroptosis acts as a danger signal to activate microglia. The results showed that the number of Iba1-positive cells increased significantly at 7–21 days after SE in the CA1, CA3, and DG regions (Figures 2I,J).

### Anti-Pyroptotic Effects of DMF in Kainic Acid-Induced Epileptic Mice

**FIGURE 3:**
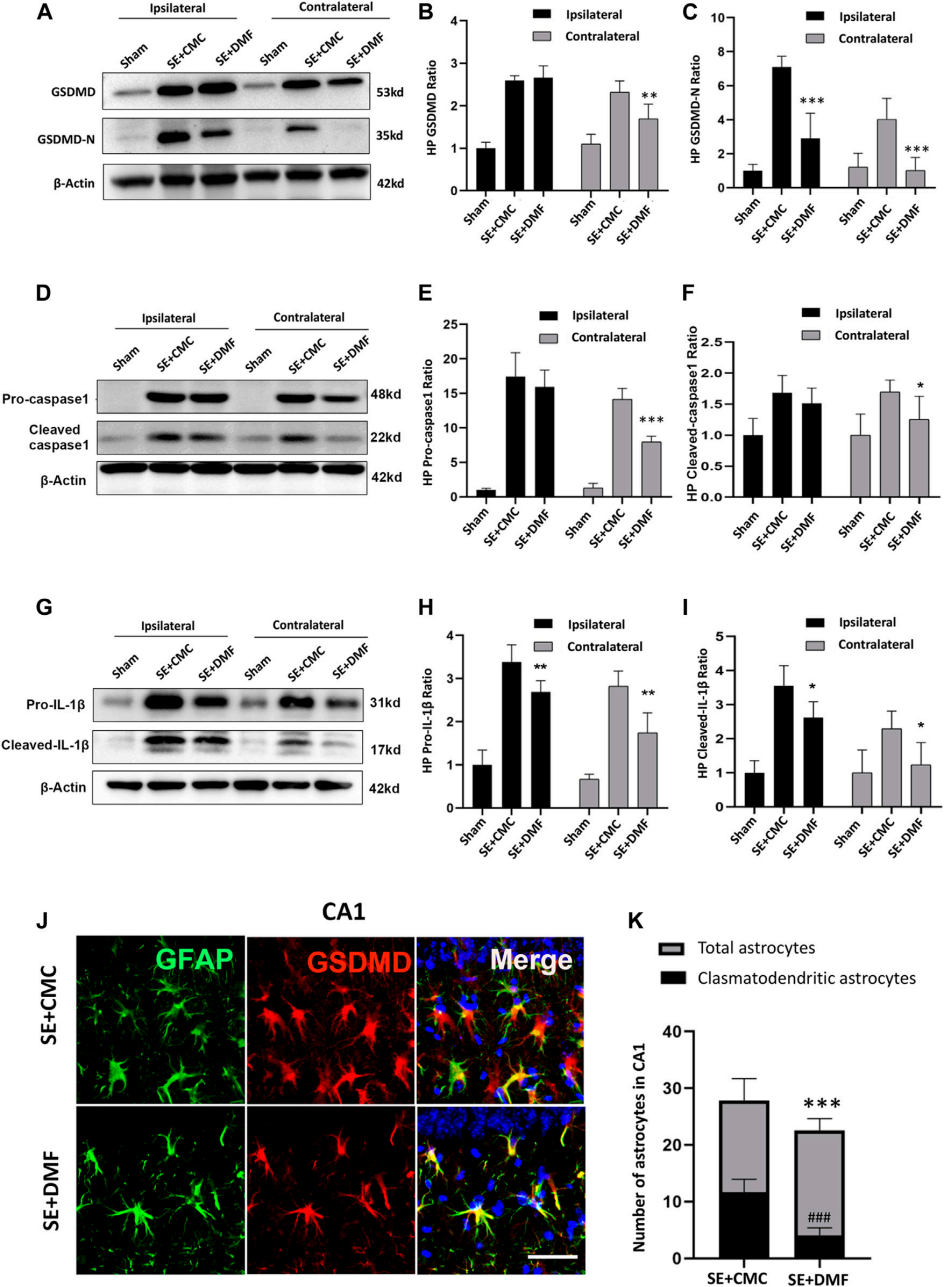
DMF intervention inhibited the expression of GSDMD-N and attenuated astrocytic clasmatodendrosis. **(A–C)** WB bands and statistical analyses of GSDMD, GSDMD-N, and β-actin proteins in the ipsilateral and contralateral hippocampus in the SE + CMC and SE + DMF groups (n = 6 in each group, asterisks represent the comparison with the SE + CMC group, *p < 0.05, **p < 0.01, and ***p < 0.001). **(D–F)** WB bands and statistical analyses of pro-caspase-1, cleaved-caspase1, and β-actin proteins in the ipsilateral and contralateral hippocampus (n = 6 in each group, asterisks indicate the comparison with the SE + CMC group, *p < 0.05, **p < 0.01, and ***p < 0.001). **(G–I)** WB bands and statistical analyses of pro-IL-1β, cleaved–IL-1β, and β-actin proteins in the ipsilateral and contralateral hippocampus (n = 6 in each group, asterisks indicate the comparison with the SE + CMC group, *p < 0.05, **p < 0.01, and ***p < 0.001). **(J)** Microphotographs of GSDMD (red) and GFAP (green) staining in the CA1 region of the hippocampus in the SE + CMC and SE + DMF groups. **(K)** Statistical analyses of the number of GSDMD-positive clasmatodendritic astrocytes and total astrocytes in the CA1 region in the SE + CMC and SE + DMF groups (n = 3 in each group, asterisks represents the total astrocytes in comparison with the sham group, ***p < 0.001; well number represents the clasmatodendritic astrocytes in comparison with the sham group, ###*p* < 0.001).

**FIGURE 4:**
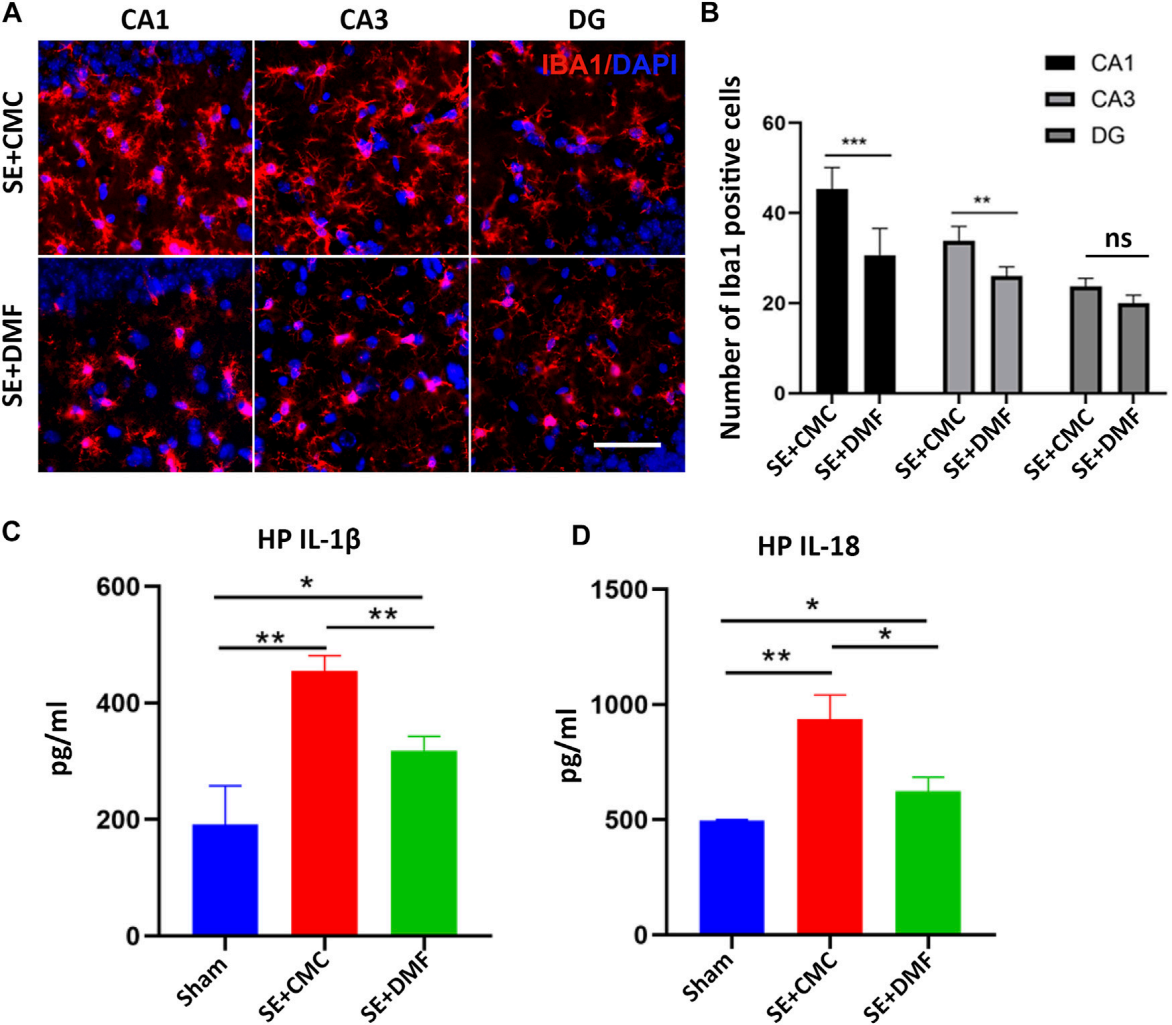
DMF intervention inhibited the activation of microglia and the expression of inflammatory factors. **(A)** Microphotographs of Iba1 (red) staining in the CA1, CA3, and DG regions of the hippocampus in the SE + CMC and SE + DMF groups. **(B)** Statistical analyses of the number of Iba1-positive cells in the CA1, CA3, and DG regions in the SE + CMC and SE + DMF groups. **(C)** Relative expression of IL-1β in the ipsilateral hippocampus based on ELISA. **(D)** Relative expression of IL-18 in the ipsilateral hippocampus based on ELISA. (n = 3 in each group, *p < 0.05, **p < 0.01, and ***p < 0.001).

To assess the anti-pyroptosis effect of DMF, WB was performed to analyze the expression of the executor GSDMD-N, between the DMF intervention group and the vehicle group at 7 days after SE, because an overtly upregulated expression of the GSDMD-N fragment was observed at this time point. The WB results showed that DMF intervention significantly reduced the expression of the GSDMD-N, although significant inhibition of full-length GSDMD was not observed in the ipsilateral hippocampus. In the contralateral hippocampus, DMF exerted obvious inhibitory effects on both the full-length GSDMD and GSDMD-N (Figures 3A–C). DMF significantly reduced the expression level of pro-caspase-1 and cleaved-caspase1 in the contralateral hippocampus, but had no obvious effect on the ipsilateral hippocampus (Figures 3D–F). The expression of pro-IL-1β and cleaved-IL-1β were significantly reduced in both the ipsilateral and contralateral hippocampus after DMF intervention (Figures 3G–I). The immunofluorescence assay showed that DMF intervention significantly attenuated the number of clasmatodendritic astrocytes and total astrocytes in the CA1 area of the hippocampus (Figures 3J,K). However, a significant influence on the number and morphology of astrocytes was not observed in the CA3 and DG regions after DMF intervention (Supplementary Figure S3). In addition, the number of Iba1-positive microglia was significantly reduced in the CA1 and CA3 areas of the hippocampus after DMF intervention (Figures 4A,B). The ELISA results also verified the inhibitory effect of DMF on inflammatory factors, including IL-1β and IL-18 (Figures 4C,D).

### DMF Reduced the Severity of Seizures in Kainic Acid-Induced Epileptic Mice

**FIGURE 5:**
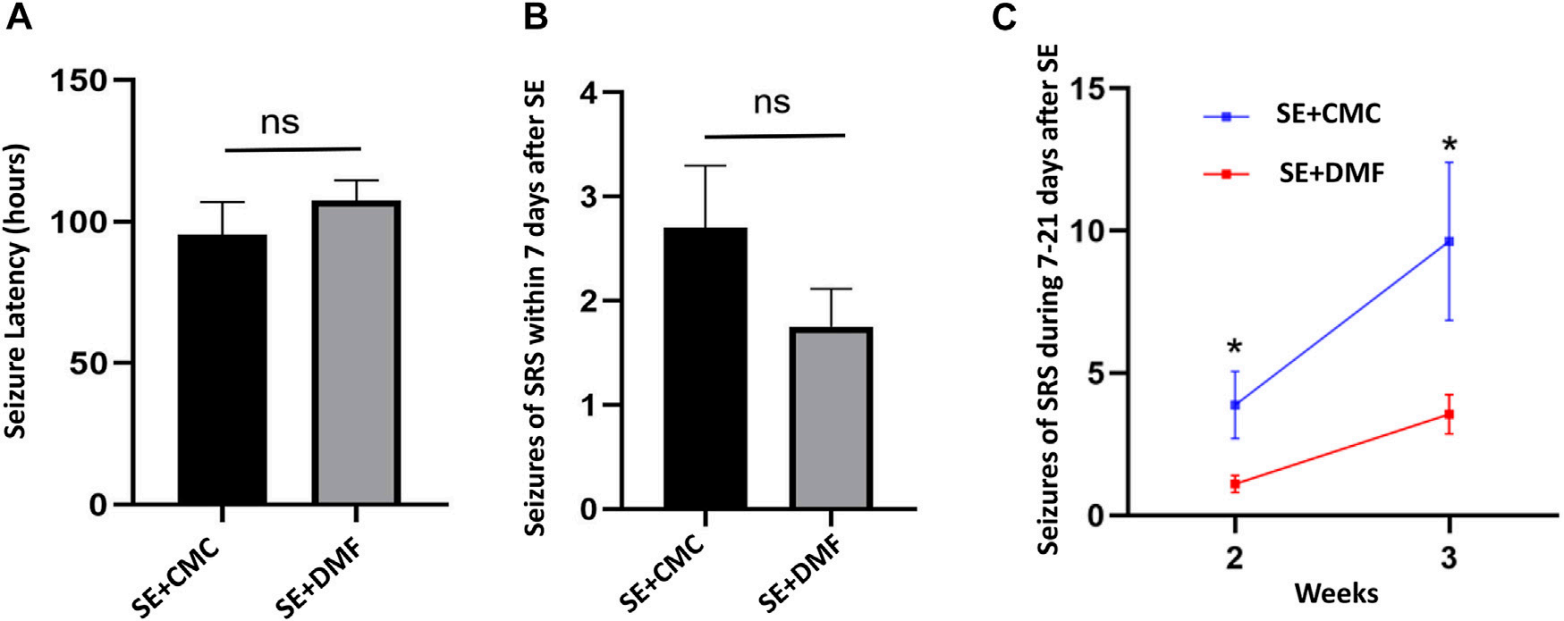
DMF intervention attenuated SRSs from 7 to 21 days after kainic acid-induced SE. **(A)** Significant differences were not observed in the latency of the first SRS within 7 days after SE between the two groups **(**n = 10 in the SE + CMC group, n = 8 in the SE + DMF group). **(B)** Significant differences were not observed in the number of SRSs within 7 days after SE between the two groups **(**n = 10 in the SE + CMC group, n = 8 in the SE + DMF group). **(C)** Number of SRSs within 7–14 days and 14–21 days post-SE significantly decreased in the SE + DMF group **(**n = 8 in the SE + CMC group, n = 9 in the SE + DMF group).

A total of 35 mice were monitored for 7 consecutive days after SE, with 18 mice in the SE + CMC group and 17 mice in the SE + DMF group. Of the 35 mice, 5 (2/18 in SE + CMC and 3/17 in SE + DMF) died after SE, while 12 mice (6/18 in the SE + CMC group and 6/17 in the SE + DMF group) without spontaneous recurrent seizures (SRSs) were excluded from the analysis within 7 days after SE. SE was induced after injection of kainic acid in the hippocampus; it generally lasted for several hours and then entered the incubation period, with SRSs appearing after several days. The latency time of the first SRSs after kainic acid injection was 95.6 ± 11.35 h and 107.5 ± 7.23 h in the SE + CMC and SE + DMF groups, respectively. No significant differences were observed in the latency of the first SRSs between the two groups (Figure 5A). The number of spontaneous seizures greater than or equal to Racine stage 4 was counted in 12 h per day (8:00–14:00 and 20:00–02:00). The number of SRSs within 7 days was 2.7 ± 0.60 and 1.75 ± 0.37 in the SE + CMC and SE + DMF groups, respectively. The number of SRSs within 7 days tended to decrease, although no significant differences were observed between the SE + CMC and SE + DMF groups (Figure 5B).

Altogether, 24 mice were used to analyze the number of SRSs 7–21 days after SE, with 12 mice each in the SE + CMC and SE + DMF groups. Out of the 24 mice, 2 (1/12 in the SE + CMC group and 1/12 in the SE + DMF group) died after SE, while 5 mice (3/12 in the SE + CMC group and 2/12 in the SE + DMF group) without SRSs were excluded from the analysis 7–21 days post-SE. The SRSs that occurred within 7–14 days and 14–21 days post-SE were significantly decreased in the SE + DMF group (Figure 5C).

## Discussion

The present study investigated the involvement of pyroptosis in epilepsy in kainic acid-induced epileptic mice. We provide the first evidence to show that the expression of GSDMD and GSDMD-N were increased after SE induced by the injection of kainic acid in the hippocampus, and that GSDMD was co-labeled with astrocytes. Seven days and 21 days after SE, astrocytes showed typical clasmatodendrosis in the CA1 region with strong GSDMD expression and was accompanied by reactivated microglia. DMF significantly inhibited the expression of GSDMD-N and inflammatory factors, including IL-1β and IL-18, attenuated astrocytic clasmatodendrosis and microglial activation, and decreased SRSs at 7–21 days after SE.

Our study found that pyroptosis-related proteins were significantly increased in both ipsilateral and contralateral hippocampus after SE. However, different tendency and expression levels of pyroptosis-related proteins were shown between two sides of the hippocampus. We propose that it may be related to the construction of the epileptic mouse model by injecting kainic acid into unilateral hippocampus. In the ipsilateral hippocampus (kainic acid injected hippocampus), kainic acid induced the formation of epileptic foci, which was the origin of epileptic discharge. The epileptic discharges from the ipsilateral hippocampus propagate to the contralateral hippocampus and caused seizures, which may be an important reason for the changes of proteins in the contralateral hippocampus. Therefore, the expression level and duration of epilepsy-related proteins were usually higher in the ipsilateral hippocampus than in the contralateral hippocampus. Meanwhile, this is also the possible reason for the subtle differences in response to DMF between two sides of the hippocampus.

The pore-forming protein GSDMD-N was recently discovered as a direct mediator of pyroptosis. Previous studies have found that GSDMD is highly expressed in liver, lung, and colon tissues, but is poorly expressed in the central nervous system under physiological conditions (Li et al., 2019). We found that GSDMD was only marginally expressed in the hippocampus of the sham group, which is consistent with previous studies. However, we found that the expression of GSDMD in the hippocampus was significantly increased after SE, suggesting the importance of this protein for further research.

Caspase-1 induces pyroptosis in GSDMD-sufficient cells but induces apoptosis in GSDMD-deficient cells (Tsuchiya et al., 2019). Therefore, cells expressing GSDMD are a prerequisite for pyroptosis. Li. J observed a time-dependent increase in the expression of pyroptosis-associated proteins in a cerebral ischemia model and found that GSDMD was mainly co-labeled in NeuN-positive cells (neurons) (Li et al., 2020). McKenzie et al. reported that GSDMD was detected within Iba-1 + microglia and GST-pi + oligodendrocytes in the lesions of patients with active multiple sclerosis (McKenzie et al., 2018). However, in contrast to previous studies, we found that GSDMD was co-labeled in astrocytes in kainic acid-induced epileptic mice. Astrocytes are the most abundant cell type in the central nervous system and are essential to protect neurons from becoming hyperexcitable (Verhoog et al., 2020). Glutamate is one of the most common excitatory amino acids in the central nervous system, and rapidly clearing excess glutamate is the key to inhibiting excessive neuronal excitement (Fonnum, 1984). However, only a small amount of glutamate released from the neuronal presynaptic membrane is taken up by the postsynaptic membrane of neurons; most of the glutamate is taken up by the glutamate transporters, excitatory amino acid transporter 1 (EAAT-1) and excitatory amino acid transporter 2 (EAAT-2), which are expressed on the astrocyte membrane to maintain a low concentration of extracellular glutamate (Mahmoud et al., 2019). Therefore, we speculate that GSDMD staining in astrocytes may be related to the important role played by astrocytes in the pathophysiological mechanism of epilepsy.

Similar to previous reports, we found damaged astrocytes characterized by clasmatodendrosis after status epilepticus (Kang et al., 2006; Kim et al., 2011; Ryu et al., 2011; Kim et al., 2015). However, we provide the first evidence to show that clasmatodendric astrocytes in the CA1 region are significantly co-expressed with GSDMD after kainic acid-induced SE, which suggests that GSDMD-mediated pyroptosis may be involved in astrocytic clasmatodendrosis. Compared with apoptosis and autophagy, pyroptosis is a form of cell death with proinflammatory immune properties (Broz et al., 2020). Pyroptosis of astrocytes not only impairs the function of astrocytes, but also induces the accumulation of surrounding immune cells and further aggravates the inflammatory response. In our study, we found that the number of clasmatodendric astrocytes increased at 7–21 days after SE, which was simultaneously accompanied by activated microglia. In addition, DMF inhibited the expression of GSDMD-N and attenuated astrocytic clasmatodendrosis and microgliosis. In our opinion, our results are consistent with the hypothesis that astrocyte pyroptosis triggers an excessive immune inflammatory response. Furthermore, we speculated that DMF may play a role in attenuating astrocytic clasmatodendrosis in the following two ways. On one hand, DMF directly inhibits the expression of GSDMD-N in the contralateral hippocampus; On the other hand, DMF inhibits the expression of GSDMD-N in the ipsilateral hippocampus, resulting in a reduction in the generation and propagation of epileptic discharges, and alleviation of seizures, which attenuates astrocytic clasmatodendrosis as well as some inflammation-related molecules in the contralateral hippocampus. Consistent with the results of previous studies, clasmatodendritic astrocytes were only observed in CA1 region, not CA3 and DG (Kim et al., 2015). However, the specific mechanism of this phenomenon remains unclear. We speculate that it may be related to the region-specific differences of certain molecules in astrocytes. We found GSDMD-positive clasmatodendritic astrocytes in the CA1 region of the hippocampus. In addition, we found that astrocytes with intact cell structures in the CA1, CA3, and DG regions of the hippocampus also express GSDMD protein, which likely occurs for the following reasons: first, astrocytes with intact cell structures present obvious GSDMD staining but only express a very small amount of GSDMD-N protein that is insufficient to cause cell membrane rupture and morphological changes in clasmatodendrosis. We believe that this problem may be solved in the near future with the development and deployment of antibodies specifically labeled with GSDMD-N fragments. Another possible reason is the complexity of the morphological structure of astrocytes coupled with the simultaneous occurrence of damage, death, activation, and proliferation, which increases the difficulty of morphological analysis. Moreover, because of the limited resolution of the optical microscope, we may not have observed subtle morphological changes in the GSDMD-positive cells. In future research, we plan to use scanning electron microscopy to observe changes in the ultrastructure of GSDMD-positive cells and apply electrophysiological analysis technology to analyze the changes in GSDMD-positive cell function.

Inhibition of GSDMD-N is the key to inhibiting pyroptosis. Dimethyl fumarate (DMF) is a fumaric acid ester that was approved in 2013 for the treatment of multiple sclerosis (Gold et al., 2012; Linker and Gold, 2013) and has shown neuroprotective effects in Parkinson’s disease (Campolo et al., 2017) and cerebral hypoxic-ischemic brain damage (Liu et al., 2019). A recent study found that DMF reacts with GSDMD at critical cysteine residues to form S (2-succinyl)-cysteine, and the succination of GSDMD blocks its progression, oligomerization, cytokine release, and cell death (Humphries et al., 2020). DMF maily inhibit the formation of GSDMD-N, but the inhibition on full-length GSDMD was not obvious. Our study is the first to demonstrate the inhibitory effect of DMF on the formation of GSDMD-N in a kainic acid-induced SE model. We found that DMF inhibited astrocyte pyroptosis after SE and alleviated SRSs during the chronic phase. However, whether other pathways contribute to antiepileptic effects remains unclear and should be further investigated. Previous studies have found that DMF acts as an antioxidative stress and anti-inflammatory agent by modulating the nuclear factor-κB (NF-κB)/nuclear transcription factor, which is related to the NF-E2 (Nrf-2) signaling pathway (Campolo et al., 2017). Moreover, DMF-induced Nrf2 expression has been reported to suppress the NF-κB-mediated pathway, which has been shown to play an anti-neuroinflammatory and apoptotic role in a pentylenetetrazol-induced kindling rat model (Singh et al., 2019). However, recent studies have found that GSDMD-mediated pyroptosis is significantly inhibited by activating the Nrf2 signaling pathway and plays a protective role in other diseases (Pang et al., 2020; Ran et al., 2020). The previously discovered role of the Nrf2-regulated oxidative stress response may involve complicated interactions with the newly discovered GSDMD-mediated pyroptosis. In future studies, we plan to use high-throughput sequencing and proteomics to clarify the specific molecular mechanism underlying the antiepileptic effect of DMF. In addition, we intend to apply more specific intervention techniques, such as siRNA and gene knockout to clearly identify the effect of GSDMD-mediated pyroptosis in epilepsy.

The present study has some limitations. First, the specific mechanism underlying the upregulation of GSDMD expression after SE has not been clarified in our study and is worthy of further research. Second, although we found that GSDMD-positive cells with significant morphological changes are accompanied by activated immune cells, direct evidence of pyroptosis is still lacking, which is a common difficulty currently seen in *in vivo* studies. Third, our preliminary results merit further verification in patients with epilepsy.

Collectively, our results showed that GSDMD-mediated pyroptosis is involved in the mechanism of kainic acid-induced seizures and is associated with astroglial damage induced by seizures. DMF alleviated the severity of seizures and astrocytic clasmatodendrosis in the CA1 region by inhibiting the expression of GSDMD-N. We suggest that GSDMD-N may be a new target for antiepileptic therapy and that DMF might represent a promising treatment strategy for epilepsy and multiple sclerosis.

## Data Availability

The original contributions presented in the study are included in the article/Supplementary Material, further inquiries can be directed to the corresponding authors.

## Supplementary Material

Supplementary Figure S1 Obvious GFAP loss in the ipsilateral hippocampus. **(A)** Images of GFAP (green)/GSDMD (red) staining in coronal hippocampal slices at bregma = −2.06 from 7 days post kainic acid-injected mice. The CA1 and CA3 regions of the ipsilateral hippocampus showed a large amount of GFAP loss (white arrowheads in lower right panels). **(B)** Images of GFAP (green)/GSDMD (red) staining in coronal hippocampal slices at bregma = −2.70 from 7-days post kainic acid-injected mice. The ipsilateral hippocampus showed more obvious GFAP loss in the CA1 and CA3 regions (white arrowheads in lower right panels) (bar = 200 µm).

Supplementary Figure S2 Astrocytes without clasmatodendrosis co-labeled with GSDMD at the CA3 and DG regions after SE. **(A)** GSDMD-positive astrocytes in the CA3 area had no significant morphological changes similar to clasmatodendrosis. **(B)** GSDMD-positive astrocytes in the DG area had no significant morphological changes similar to clasmatodendrosis (bar = 50 µm).

Supplementary Figure S3 DMF had no significant influence on the astrocytes in the CA3 and DG regions. **(A)** Astrocytes without clasmatodendrosis at CA3 regions after SE. Significant differences in the number of total astrocytes in CA3 were not found between SE + CMC and SE + DMF **(B)** Astrocytes without clasmatodendrosis in DG regions after SE. Significant differences in the number of total astrocytes in the DG were not found between SE + CMC and SE + DMF (bar = 50 µm).

## Abbreviations

GSDMD, Gasdermin D; SE, Status epilepticus; DMF, Dimethyl fumarate; GSDMD-N, Gasdermin D N-terminal fragments; CMC, Carboxymethyl cellulose; SRSs, Recurrent spontaneous seizures; NLR, Nucleotide oligomerization domain like receptor; NLRP1, NLR family pyrin domain-containing 1; NLRP3, NLR family pyrin domain-containing 3; AIM2, Absent in melanoma 2; DAMPs, Damage-associated molecular patterns; HMGB1, High-mobility group box 1; ATP, Adenosine 5′-triphosphate; LPS, Lipopolysaccharide; ELISA, Enzyme-linked immunosorbent assay; EAAT-1, Excitatory amino acid transporter 1; EAAT-2, Excitatory amino acid transporter 2.

## References

[B1] Aronica E. Bauer S. Bozzi Y. Caleo M. Dingledine R. Gorter J. A. (2017). Neuroinflammatory Targets and Treatments for Epilepsy Validated in Experimental Models. Epilepsia 58 (Suppl. 3), 27–38. 10.1111/epi.13783 PMC5873599 28675563

[B2] Broz P. Pelegrín P. Shao F. (2020). The Gasdermins, a Protein Family Executing Cell Death and Inflammation. Nat. Rev. Immunol. 20 (3), 143–157. 10.1038/s41577-019-0228-2 31690840

[B3] Campolo M. Casili G. Biundo F. Crupi R. Cordaro M. Cuzzocrea S. (2017). The Neuroprotective Effect of Dimethyl Fumarate in an MPTP-Mouse Model of Parkinson's Disease: Involvement of Reactive Oxygen Species/Nuclear Factor-κB/Nuclear Transcription Factor Related to NF-E2. Antioxid. Redox Signal. 27 (8), 453–471. 10.1089/ars.2016.6800 28006954 PMC5564046

[B4] Cristina de Brito Toscano E. Leandro Marciano Vieira É. Boni Rocha Dias B. Vidigal Caliari M. Paula Gonçalves A. Varela Giannetti A. (2021). NLRP3 and NLRP1 Inflammasomes Are Up-Regulated in Patients with Mesial Temporal Lobe Epilepsy and May Contribute to Overexpression of Caspase-1 and IL-β in Sclerotic Hippocampi. Brain Res. 1752, 147230. 10.1016/j.brainres.2020.147230 33385378

[B5] Feng L. Murugan M. Bosco D. B. Liu Y. Peng J. Worrell G. A. (2019). Microglial Proliferation and Monocyte Infiltration Contribute to Microgliosis Following Status Epilepticus. Glia 67 (8), 1434–1448. 10.1002/glia.23616 31179602 PMC6559368

[B6] Fonnum F. (1984). Glutamate: a Neurotransmitter in Mammalian Brain. J. Neurochem. 42 (1), 1–11. 10.1111/j.1471-4159.1984.tb09689.x 6139418

[B7] Gold R. Kappos L. Arnold D. L. Bar-Or A. Giovannoni G. Selmaj K. (2012). Placebo-controlled Phase 3 Study of Oral BG-12 for Relapsing Multiple Sclerosis. N. Engl. J. Med. 367 (12), 1098–1107. 10.1056/NEJMoa1114287 22992073

[B8] Han C. Yang Y. Guan Q. Zhang X. Shen H. Sheng Y. (2020). New Mechanism of Nerve Injury in Alzheimer's Disease: β-amyloid-induced Neuronal Pyroptosis. J. Cel Mol Med 24 (14), 8078–8090. 10.1111/jcmm.15439 PMC7348172 32521573

[B9] Hase Y. Chen A. Bates L. L. Craggs L. J. L. Yamamoto Y. Gemmell E. (2018). Severe white Matter Astrocytopathy in CADASIL. Brain Pathol. 28 (6), 832–843. 10.1111/bpa.12621 29757481 PMC8028291

[B10] Huang X. Feng Y. Xiong G. Whyte S. Duan J. Yang Y. (2019). Caspase-11, a Specific Sensor for Intracellular Lipopolysaccharide Recognition, Mediates the Non-canonical Inflammatory Pathway of Pyroptosis. Cell Biosci 9, 31. 10.1186/s13578-019-0292-0 30962873 PMC6438033

[B11] Humphries F. Shmuel-Galia L. Ketelut-Carneiro N. Li S. Wang B. Nemmara V. V. (2020). Succination Inactivates Gasdermin D and Blocks Pyroptosis. Science 369 (6511), 1633–1637. 10.1126/science.abb9818 32820063 PMC8744141

[B12] Jiang Z. Chen J. Chen J. Lei Z. Chen H. Wu J. (2021). Anti-inflammatory Effects of Paeoniflorin Caused by Regulation of the hif1a/miR-210/caspase1/GSDMD Signaling Pathway in Astrocytes: a Novel Strategy for Hypoxia-Induced Brain Injury in Rats. Immunopharmacology and Immunotoxicology 43, 410–418. 10.1080/08923973.2021.1924194 34114917

[B13] Kang T. C. Kim D. S. Kwak S. E. Kim J. E. Won M. H. Kim D. W. (2006). Epileptogenic Roles of Astroglial Death and Regeneration in the Dentate Gyrus of Experimental Temporal Lobe Epilepsy. Glia 54 (4), 258–271. 10.1002/glia.20380 16845674

[B14] Kayagaki N. Stowe I. B. Lee B. L. O'Rourke K. Anderson K. Warming S. (2015). Caspase-11 Cleaves Gasdermin D for Non-canonical Inflammasome Signalling. Nature 526 (7575), 666–671. 10.1038/nature15541 26375259

[B15] Kim D. S. Kim J. E. Kwak S. E. Choi K. C. Kim D. W. Kwon O. S. (2008). Spatiotemporal Characteristics of Astroglial Death in the Rat Hippocampo-Entorhinal Complex Following Pilocarpine-Induced Status Epilepticus. J. Comp. Neurol. 511 (5), 581–598. 10.1002/cne.21851 18853423

[B16] Kim J. E. Ryu H. J. Yeo S. I. Kang T. C. (2011). P2X7 Receptor Differentially Modulates Astroglial Apoptosis and Clasmatodendrosis in the Rat Brain Following Status Epilepticus. Hippocampus 21 (12), 1318–1333. 10.1002/hipo.20850 20848604

[B17] Kim J. Y. Ko A. R. Kim J. E. (2015). P2X7 Receptor-Mediated PARP1 Activity Regulates Astroglial Death in the Rat hippocampus Following Status Epilepticus. Front Cel Neurosci 9, 352. 10.3389/fncel.2015.00352 PMC4560025 26388738

[B18] Li J. Hao J. H. Yao D. Li R. Li X. F. Yu Z. Y. (2020). Caspase‐1 Inhibition Prevents Neuronal Death by Targeting the Canonical Inflammasome Pathway of Pyroptosis in a Murine Model of Cerebral Ischemia. CNS Neurosci. Ther. 26, 925–939. 10.1111/cns.13384 32343048 PMC7415206

[B19] Li S. Wu Y. Yang D. Wu C. Ma C. Liu X. (2019). Gasdermin D in Peripheral Myeloid Cells Drives Neuroinflammation in Experimental Autoimmune Encephalomyelitis. J. Exp. Med. 216 (11), 2562–2581. 10.1084/jem.20190377 31467036 PMC6829591

[B20] Linker R. A. Gold R. (2013). Dimethyl Fumarate for Treatment of Multiple Sclerosis: Mechanism of Action, Effectiveness, and Side Effects. Curr. Neurol. Neurosci. Rep. 13 (11), 394. 10.1007/s11910-013-0394-8 24061646

[B21] Liu L. Vollmer M. K. Ahmad A. S. Fernandez V. M. Kim H. Doré S. (2019). Pretreatment with Korean Red Ginseng or Dimethyl Fumarate Attenuates Reactive Gliosis and Confers Sustained Neuroprotection against Cerebral Hypoxic-Ischemic Damage by an Nrf2-dependent Mechanism. Free Radic. Biol. Med. 131, 98–114. 10.1016/j.freeradbiomed.2018.11.017 30458277 PMC6362849

[B22] Mahmoud S. Gharagozloo M. Simard C. Gris D. (2019). Astrocytes Maintain Glutamate Homeostasis in the CNS by Controlling the Balance between Glutamate Uptake and Release. Cells 8 (2), 184. 10.3390/cells8020184 PMC6406900 30791579

[B23] Maroso M. Balosso S. Ravizza T. Iori V. Wright C. I. French J. (2011). Interleukin-1β Biosynthesis Inhibition Reduces Acute Seizures and Drug Resistant Chronic Epileptic Activity in Mice. Neurotherapeutics 8 (2), 304–315. 10.1007/s13311-011-0039-z 21431948 PMC3101825

[B24] Maroso M. Balosso S. Ravizza T. Liu J. Aronica E. Iyer A. M. (2010). Toll-like Receptor 4 and High-Mobility Group Box-1 Are Involved in Ictogenesis and Can Be Targeted to Reduce Seizures. Nat. Med. 16 (4), 413–419. 10.1038/nm.2127 20348922

[B25] McKenzie B. A. Mamik M. K. Saito L. B. Boghozian R. Monaco M. C. Major E. O. (2018). Caspase-1 Inhibition Prevents Glial Inflammasome Activation and Pyroptosis in Models of Multiple Sclerosis. Proc. Natl. Acad. Sci. U S A. 115 (26), E6065–e6074. 10.1073/pnas.1722041115 29895691 PMC6042136

[B26] Pang Y. Zhang P. C. Lu R. R. Li H. L. Li J. C. Fu H. X. (2020). Andrade-Oliveira Salvianolic Acid B Modulates Caspase-1-Mediated Pyroptosis in Renal Ischemia-Reperfusion Injury via Nrf2 Pathway. Front. Pharmacol. 11, 541426. 10.3389/fphar.2020.541426 33013384 PMC7495093

[B27] Penfield W. G. (1928). Neuroglia and Microglia - the Interstitial Tissue of the central Nervous System.

[B28] Racine R. J. (1972). Modification of Seizure Activity by Electrical Stimulation. II. Motor Seizure. Electroencephalogr Clin. Neurophysiol. 32 (3), 281–294. 10.1016/0013-4694(72)90177-0 4110397

[B29] Ran X. Yan Z. Yang Y. Hu G. Liu J. Hou S. (2020). Dioscin Improves Pyroptosis in LPS-Induced Mice Mastitis by Activating AMPK/Nrf2 and Inhibiting the NF-Κb Signaling Pathway. Oxid Med. Cel Longev 2020, 8845521. 10.1155/2020/8845521 PMC7790561 33488936

[B30] Rana A. Musto A. E. (2018). The Role of Inflammation in the Development of Epilepsy. J. Neuroinflammation 15 (1), 144. 10.1186/s12974-018-1192-7 29764485 PMC5952578

[B31] Ryu H. J. Kim J. E. Yeo S. I. Kang T. C. (2011). p65/RelA-Ser529 NF-Κb Subunit Phosphorylation Induces Autophagic Astroglial Death (Clasmatodendrosis) Following Status Epilepticus. Cell Mol Neurobiol 31 (7), 1071–1078. 10.1007/s10571-011-9706-1 21598036 PMC11498587

[B32] Sanz P. Garcia-Gimeno M. A. (2020). Reactive Glia Inflammatory Signaling Pathways and Epilepsy. Int. J. Mol. Sci. 21 (11), 4096. 10.3390/ijms21114096 PMC7312833 32521797

[B33] Shi J. Gao W. Shao F. (2017). Pyroptosis: Gasdermin-Mediated Programmed Necrotic Cell Death. Trends Biochem. Sci. 42 (4), 245–254. 10.1016/j.tibs.2016.10.004 27932073

[B34] Shi J. Zhao Y. Wang K. Shi X. Wang Y. Huang H. (2015). Cleavage of GSDMD by Inflammatory Caspases Determines Pyroptotic Cell Death. Nature 526 (7575), 660–665. 10.1038/nature15514 26375003

[B35] Singh N. Saha L. Kumari P. Singh J. Bhatia A. Banerjee D. (2019). Effect of Dimethyl Fumarate on Neuroinflammation and Apoptosis in Pentylenetetrazol Kindling Model in Rats. Brain Res. Bull. 144, 233–245. 10.1016/j.brainresbull.2018.11.013 30472152

[B36] Sun Y. B. Zhao H. Mu D. L. Zhang W. Cui J. Wu L. (2019). Dexmedetomidine Inhibits Astrocyte Pyroptosis and Subsequently Protects the Brain in *In Vitro* and *In Vivo* Models of Sepsis. Cell Death Dis 10 (3), 167. 10.1038/s41419-019-1416-5 30778043 PMC6379430

[B37] Thijs R. D. Surges R. O'Brien T. J. Sander J. W. (2019). Epilepsy in Adults. Lancet 393 (10172), 689–701. 10.1016/s0140-6736(18)32596-0 30686584

[B38] Tsuchiya K. Nakajima S. Hosojima S. Thi Nguyen D. Hattori T. Manh Le T. (2019). Caspase-1 Initiates Apoptosis in the Absence of Gasdermin D. Nat. Commun. 10 (1), 2091. 10.1038/s41467-019-09753-2 31064994 PMC6505044

[B39] Verhoog Q. P. Holtman L. Aronica E. van Vliet E. A. (2020). Astrocytes as Guardians of Neuronal Excitability: Mechanisms Underlying Epileptogenesis. Front. Neurol. 11, 591690. 10.3389/fneur.2020.591690 33324329 PMC7726323

[B40] Vezzani A. Balosso S. Ravizza T. (2019). Neuroinflammatory Pathways as Treatment Targets and Biomarkers in Epilepsy. Nat. Rev. Neurol. 15 (8), 459–472. 10.1038/s41582-019-0217-x 31263255

[B41] Xia S. Zhang Z. Magupalli V. G. Pablo J. L. Dong Y. Vora S. M. (2021). Gasdermin D Pore Structure Reveals Preferential Release of Mature Interleukin-1. Nature 593 (7860), 607–611. 10.1038/s41586-021-03478-3 33883744 PMC8588876

