# Supplementary figures and images for: Inhibition of Gasdermin D-Mediated Pyroptosis Attenuates the Severity of Seizures and Astroglial Damage in Kainic Acid-Induced Epileptic Mice
Source: Front Pharmacol. 2022 Jan 28;12:751644. doi: 10.3389/fphar.2021.751644 (PMC8831916; doi:10.3389/fphar.2021.751644)

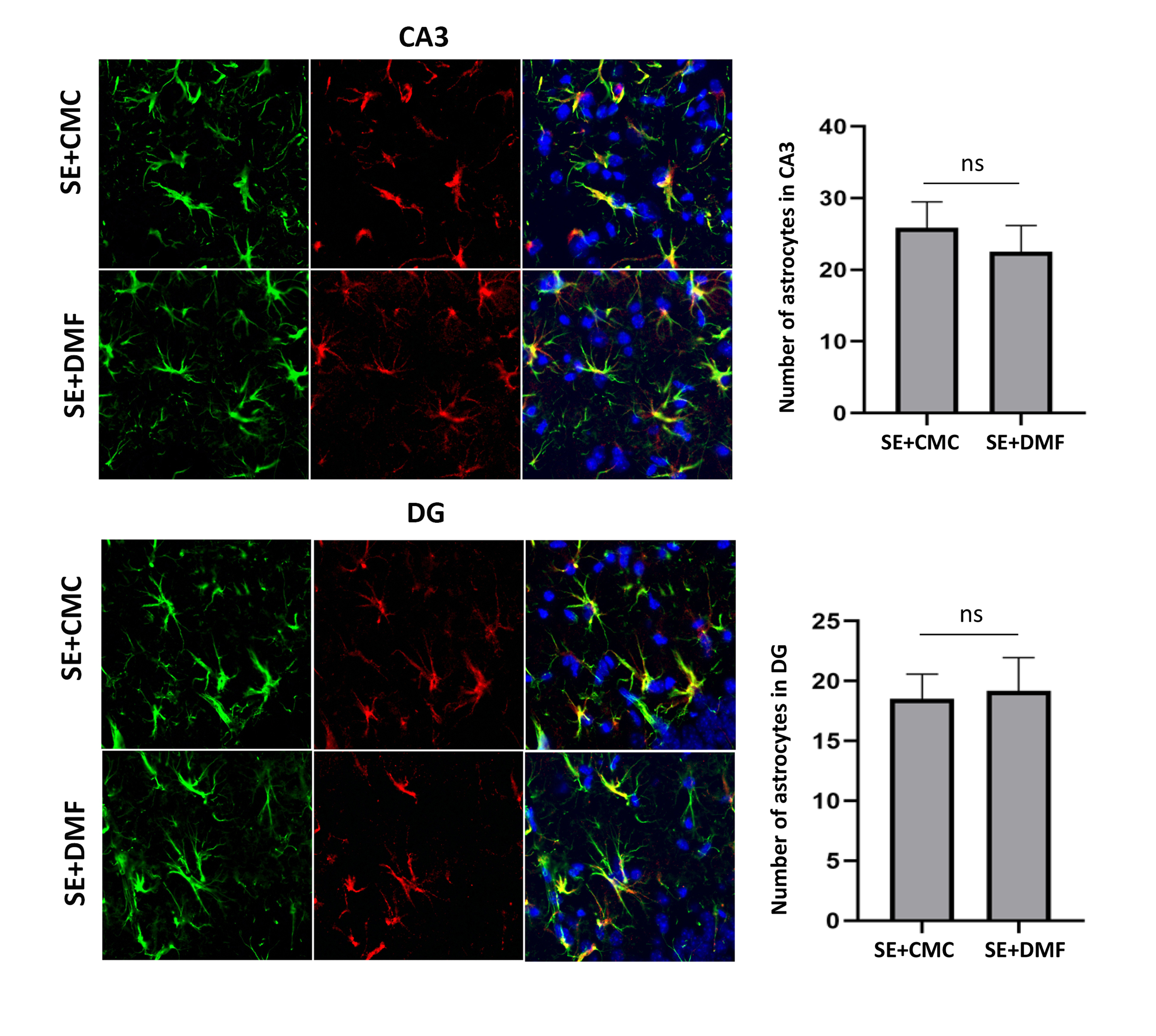

Supplement: Supplementary file 1 [file Image3.TIF]

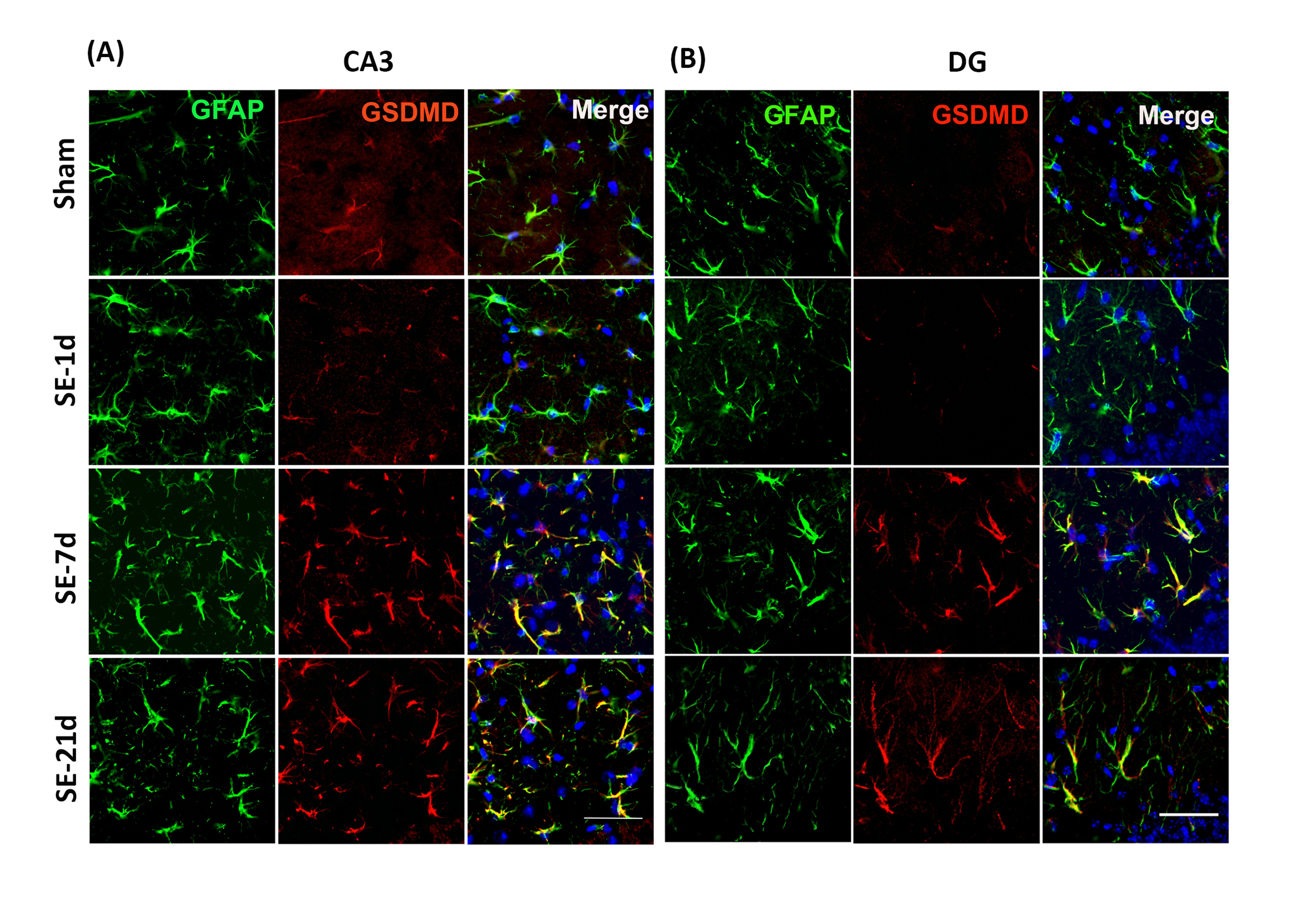

Supplement: Supplementary file 2 [file Image2.TIF]

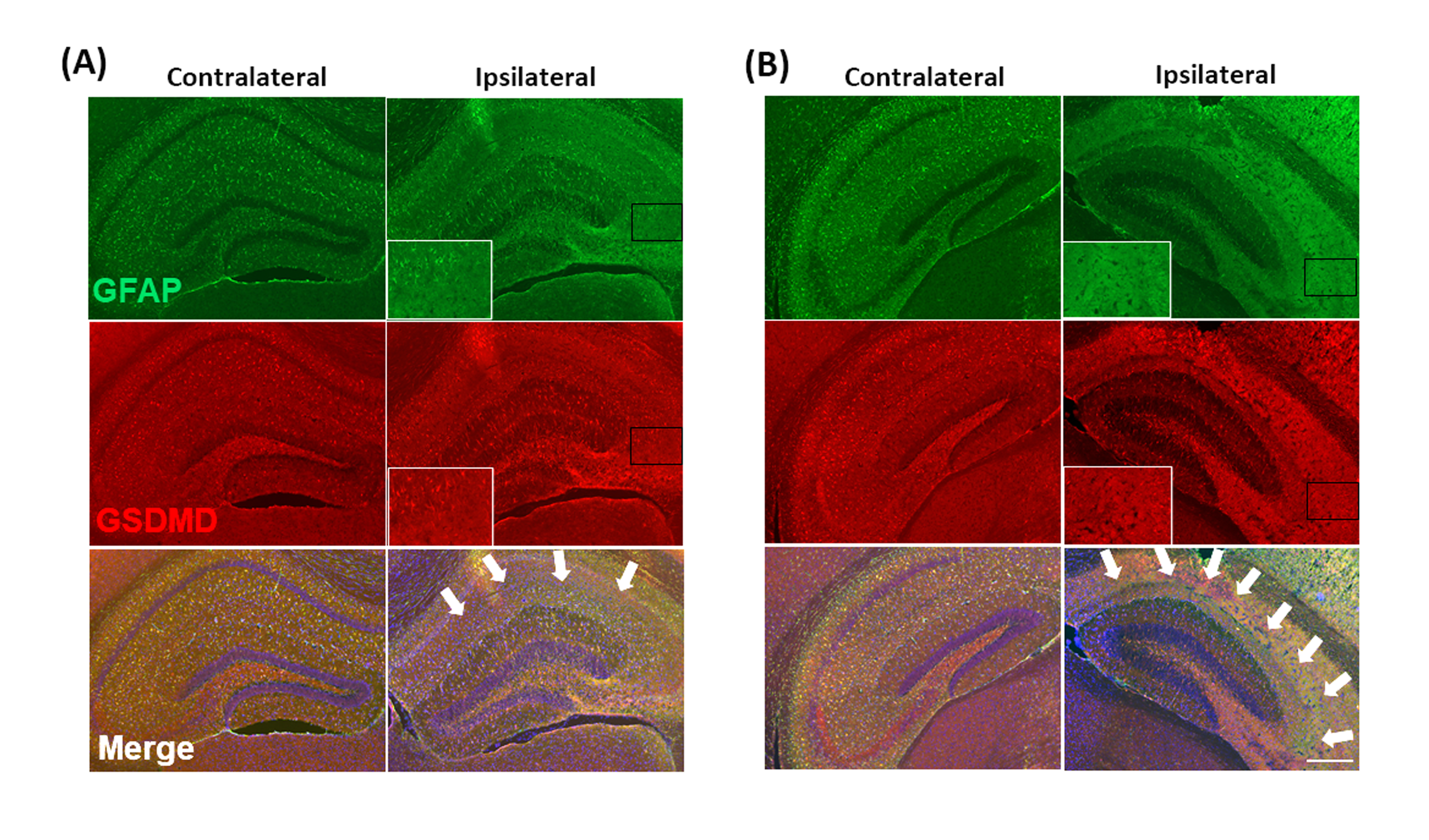

Supplement: Supplementary file 3 [file Image1.TIF]
