# Supplementary material for: Inhibition of Gasdermin D-Mediated Pyroptosis Attenuates the Severity of Seizures and Astroglial Damage in Kainic Acid-Induced Epileptic Mice
Source: Front Pharmacol. 2022 Jan 28;12:751644. doi: 10.3389/fphar.2021.751644 (PMC8831916; doi:10.3389/fphar.2021.751644)
Supplement: Supplementary file 4 [file DataSheet1.docx]

**Figure 1A**

1. (B)


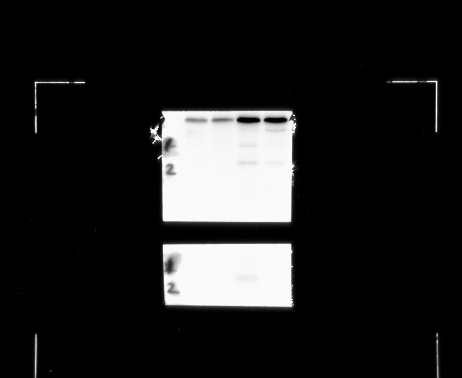

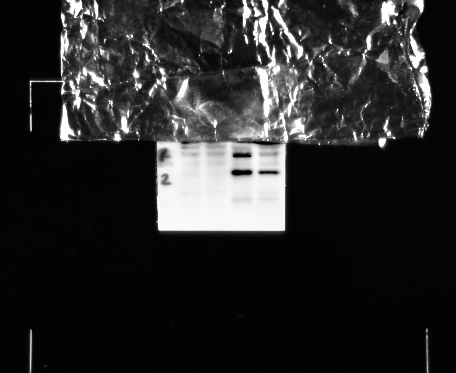


(C)


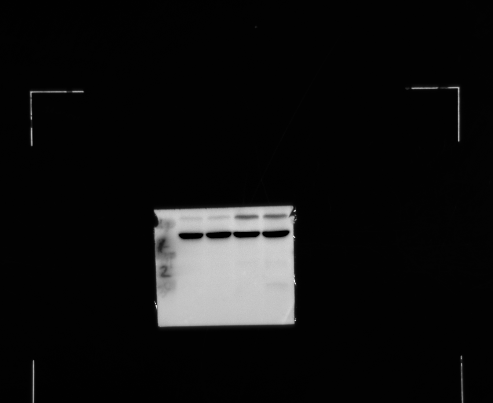


(A) The unedited blot demonstrated the full-length GSDMD in figure 1A.

(B) The unedited blot demonstrated the GSDMD-N in figure 1A.

(C) The membrane of (A) and (B) further incubated with the β-actin antibody. The unedited blot demonstrated the β-Actin in figure 1A

**Figure 1B**

1. （B）


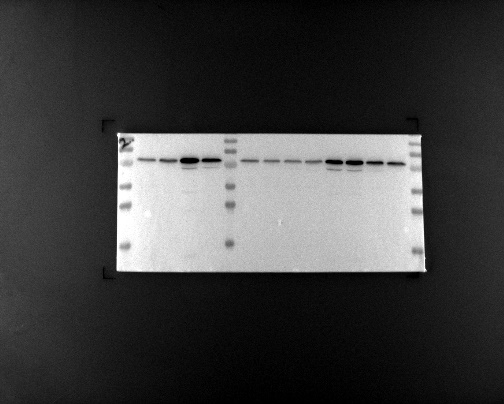

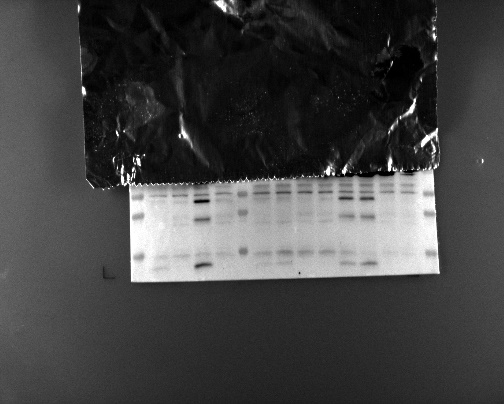


(C)


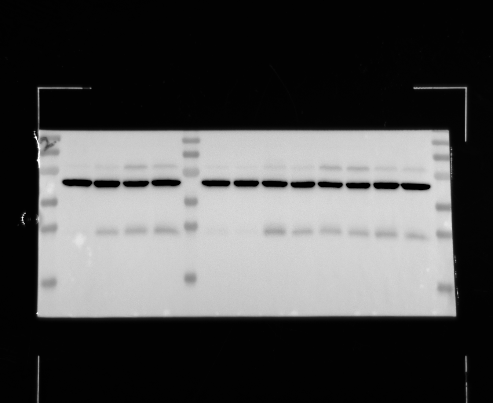


(A) The unedited blot demonstrated the full-length GSDMD in figure 1B.

(B) The unedited blot demonstrated the GSDMD-N in figure 1B.

(C) The membrane of (A) and (B) further incubated with the β-actin antibody. The unedited blot demonstrated the β-Actin in figure 1B

**Figure1G**

1. (B)


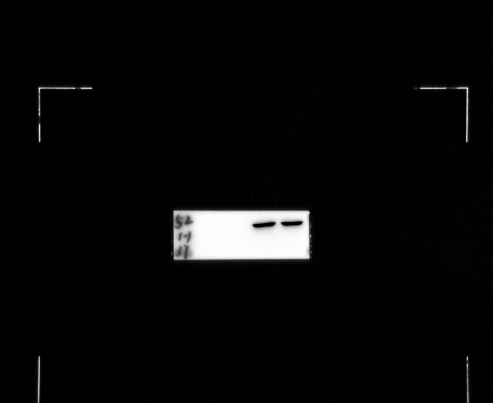

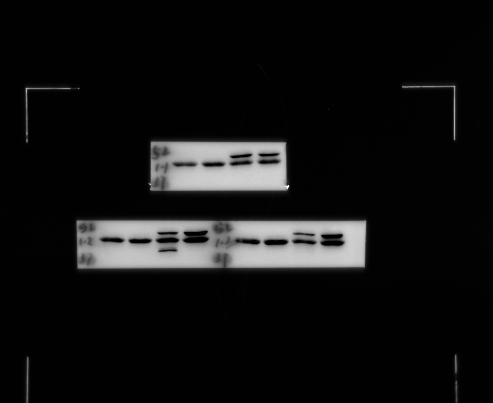
V

(C) (D)


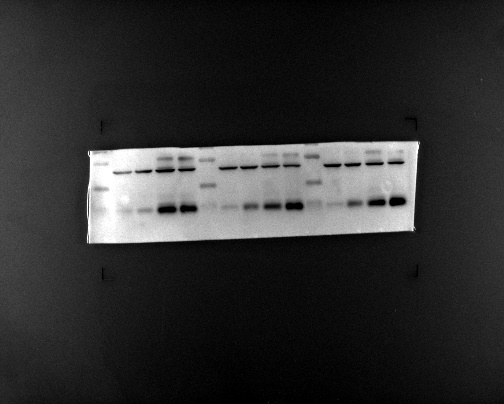

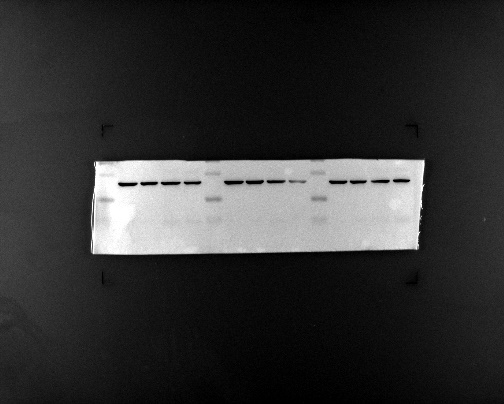


(A) The unedited blot demonstrated the Caspase1 in figure 1G.

(B) The membrane of (A) further incubated with the β-actin antibody. The unedited blot demonstrated the β-Actin in figure 1G

(C) The unedited blot demonstrated the IL-1β and β-Actin in figure 1G. the lane was IL-1β in figure 1G.

(D) The membrane of (C) further incubated with the β-actin antibody. The unedited blot demonstrated the β-Actin in figure 1G

**Figure 1H**

1. (B)


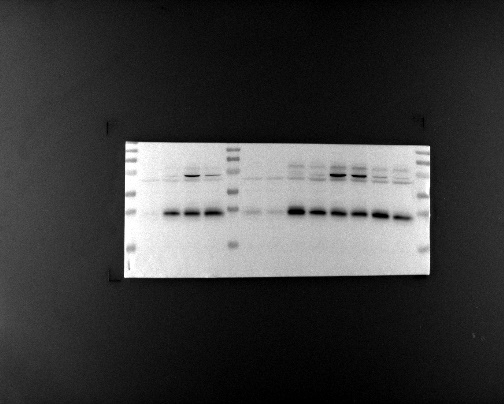

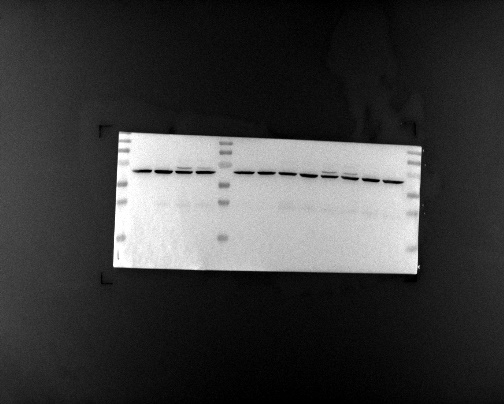


(C ) （D）


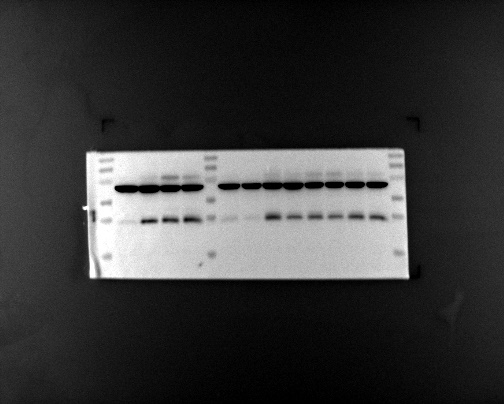

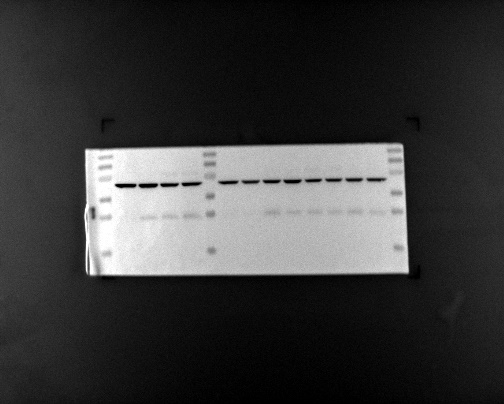


(A) The membrane incubated with the caspase1 and IL-1β antibody，the lane was caspase1 in figure 1H.

(B) The membrane of (A) further incubated with the β-actin antibody. The unedited blot demonstrated the β-Actin in figure 1H

(C) The membrane incubated with the IL-1β and β-actin antibody，the lane was IL-1β in figure 1H.

(D) The membrane (C) further incubated with the β-actin antibody. The unedited blot demonstrated the β-Actin in figure 1H.

**Figure3A**

1. (B)


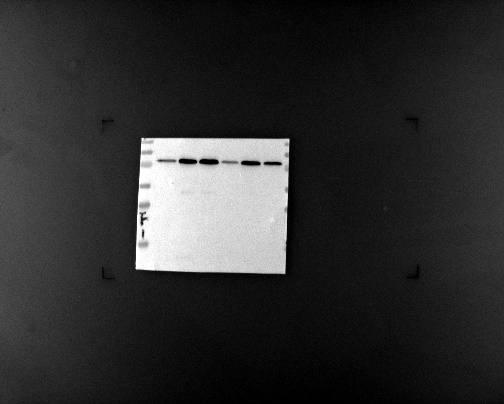

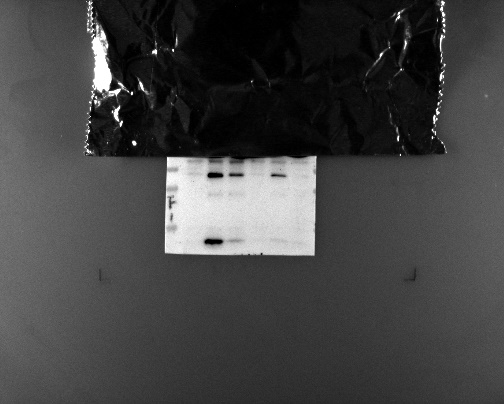


(C)


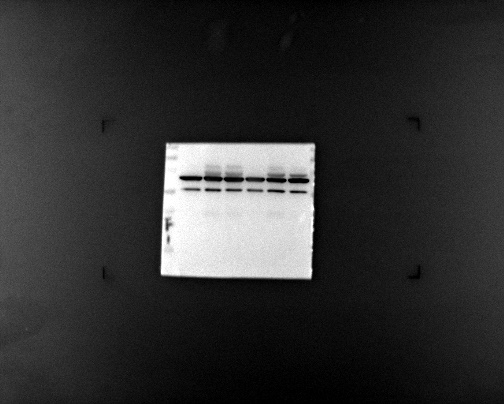


(A) The unedited blot demonstrated the full-length GSDMD in figure 3A

(B) The unedited blot demonstrated the GSDMD-N in figure 3A.

(C) The membrane of (A) and (B) further incubated with the β-actin antibody. The unedited blot demonstrated the β-Actin in figure 3A.

**Figure3D**

1. (B)


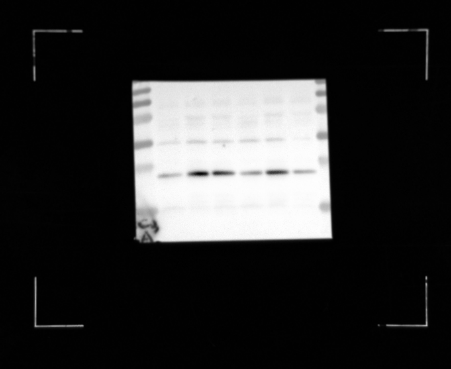

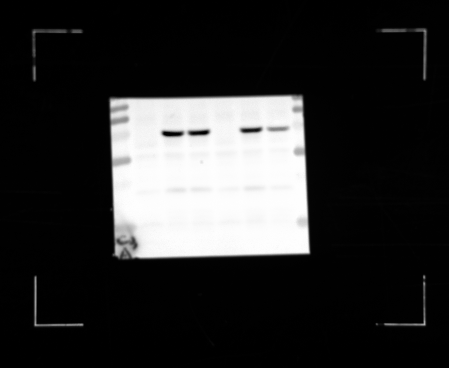


(C )


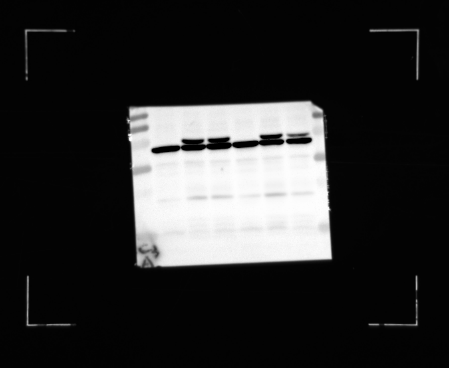


(A) The unedited blot demonstrated the cleaved-caspase1 in figure 3D.

(B) The membrane of (A) further incubated with the pro-caspase1 antibody. The unedited blot demonstrated the pro-caspase1 in figure 3D

(C) The membrane of (A) further incubated with the β-actin antibody. The unedited blot demonstrated the β-Actin in figure 3D.

**Figure3G**

1. (B)


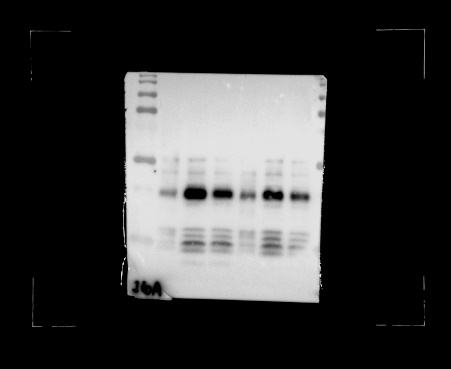

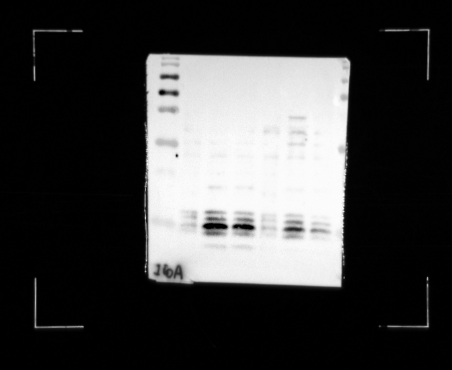


(C )


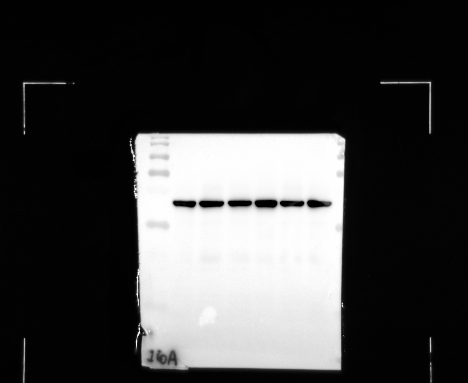


(A) The unedited blot demonstrated the cleaved-IL1β in figure 3G.

(B) The membrane of (A) further incubated with the pro- IL1β antibody. The unedited blot demonstrated the pro-caspase1 in figure 3G

(C) The membrane of (A) further incubated with the β-actin antibody. The unedited blot demonstrated the β-Actin in figure 3G.

**Figure 2A-C**


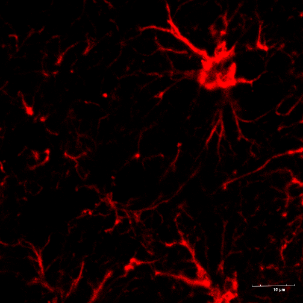
(A)


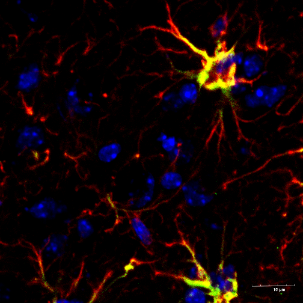

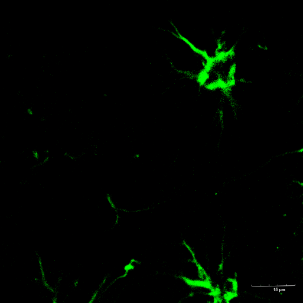


(E)

1.
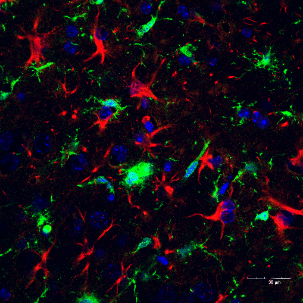
 (C)


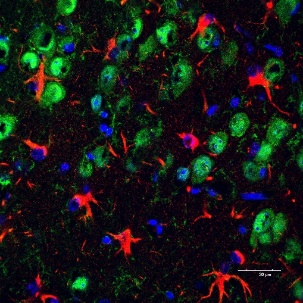


1. The original microscopy images in Figure 2A
2. The original microscopy images in Figure 2B
3. The original microscopy images in Figure 2C

**Figure 2D**

(A)


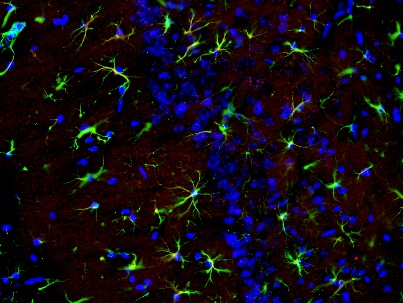

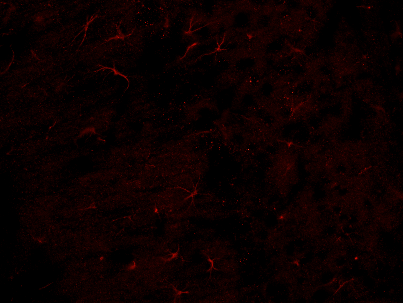

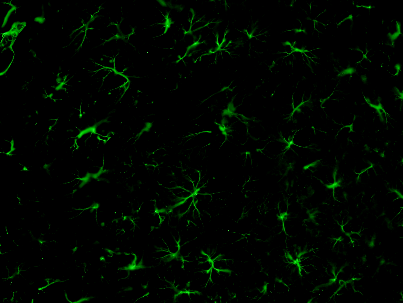


（C）(D) (E) The original microscopy images of Figure 2A


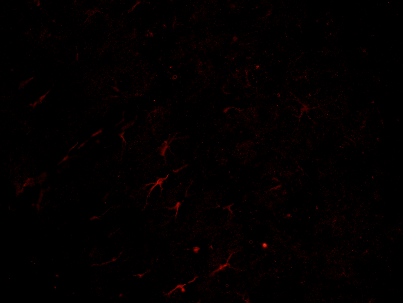

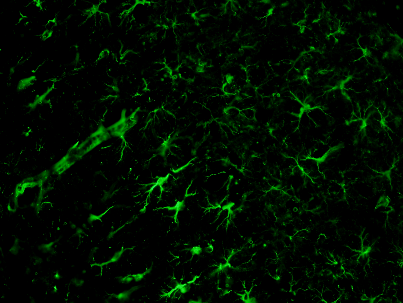
(B)


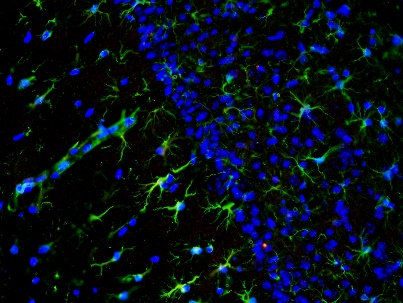


(C)


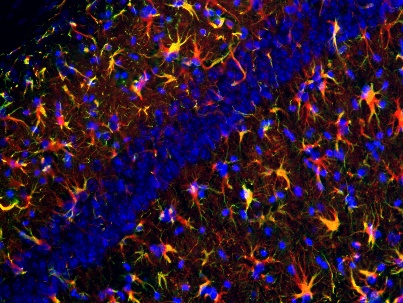

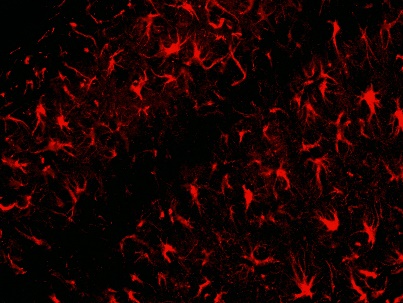

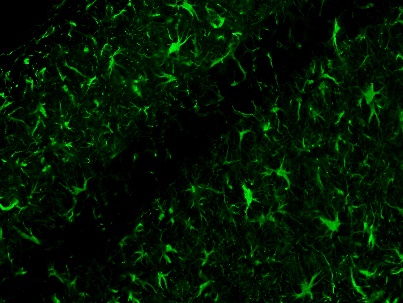


(D)


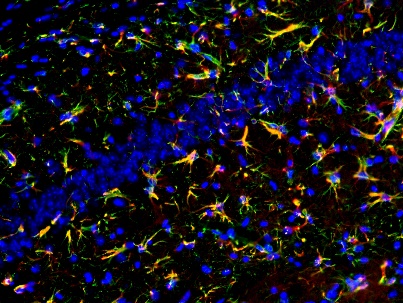

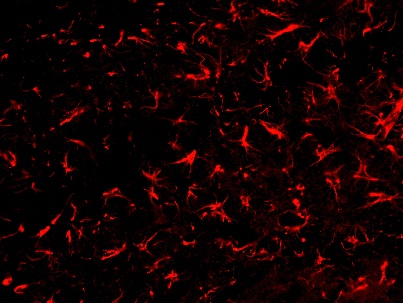

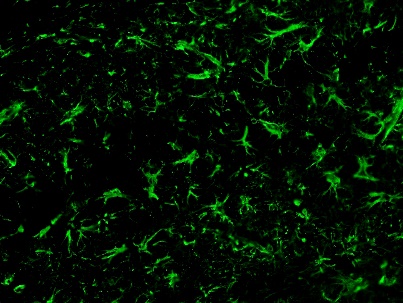


1. The original microscopy images of the Sham group in Figure 2D
2. The original microscopy images of the SE-1d group in Figure 2D
3. The original microscopy images of the SE-7d group in Figure 2D
4. The original microscopy images of the SE-21d group in Figure 2D

**Figure 2E**

(A)


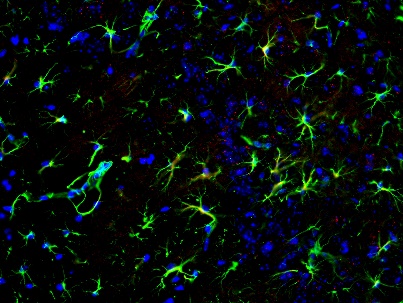

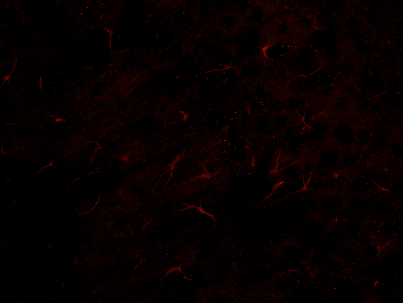

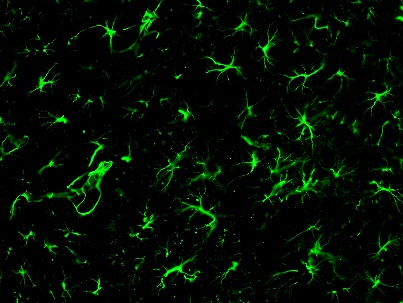


(B)


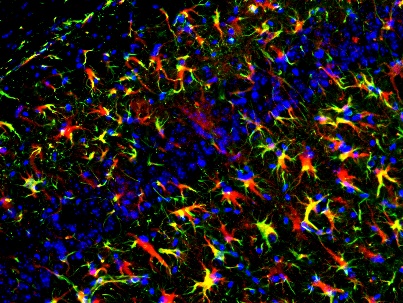

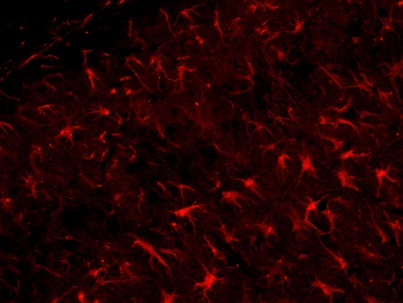

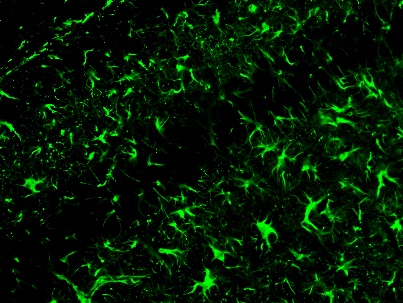


(C)


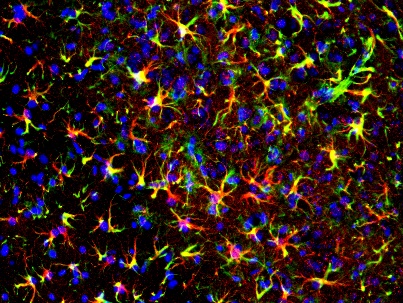

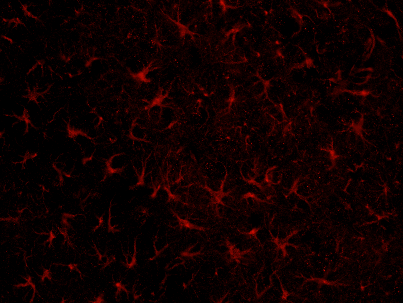

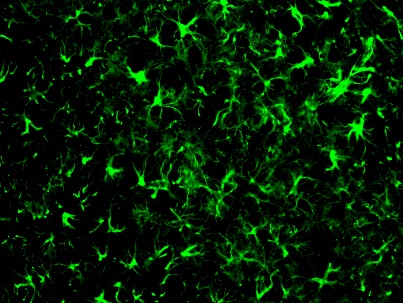


1. The original microscopy images of the Sham group in Figure 2E.
2. (C) The original microscopy images of the SE-7d in Figure 2E. The group of SE+CMC also could represent 7 days after SE to show the typical clasmatodendritic astrocytes.

**Figure 2I**

（A）


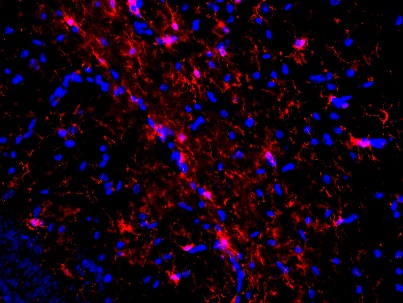

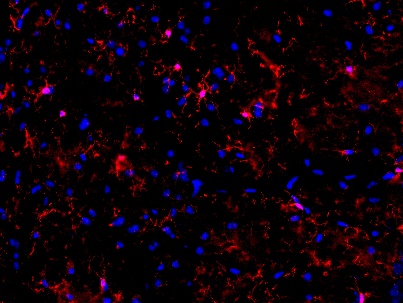

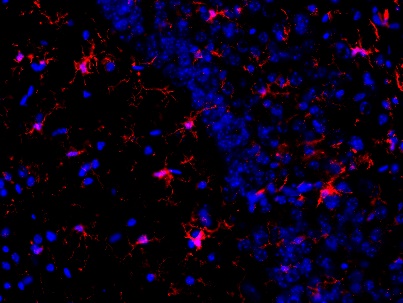


(B)


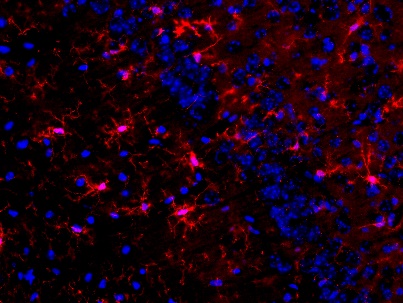

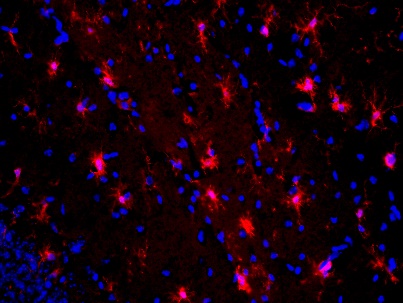

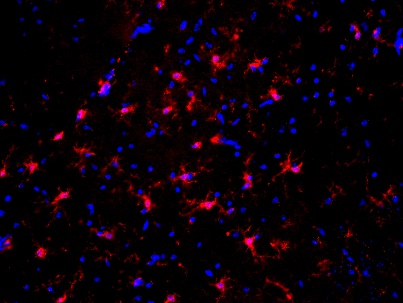
(C)


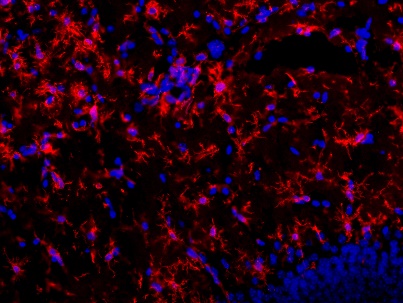

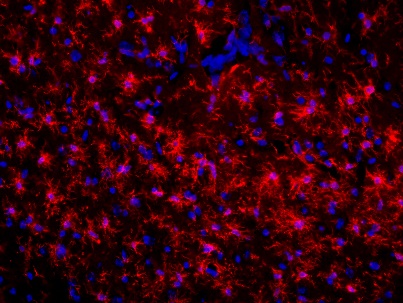

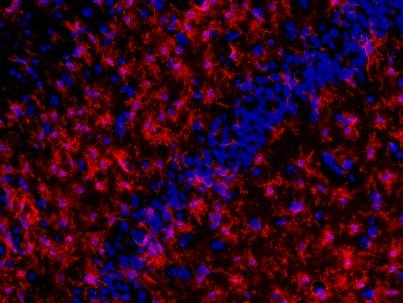


(D)


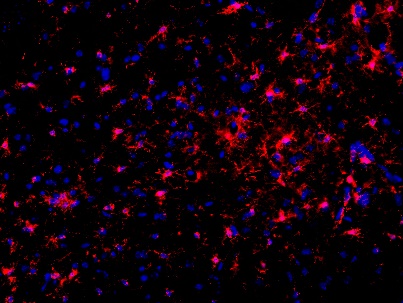

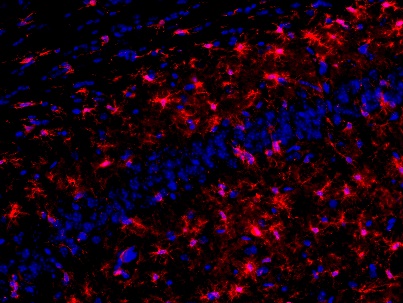

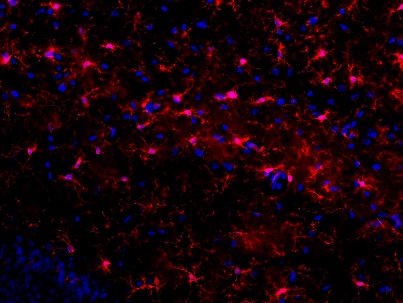


(A) The original microscopy images of the Sham group in Figure 2I

(B) The original microscopy images of the SE-1d group in Figure 2I

(C) The original microscopy images of the SE-7d group in Figure 2I

(D) The original microscopy images of the SE-21d group in Figure 2I

**Figure 3J**

（A）


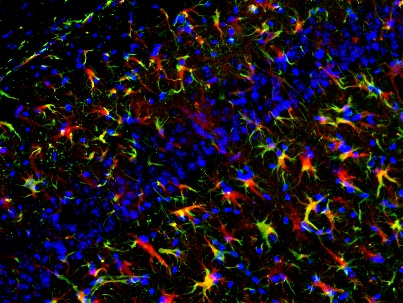

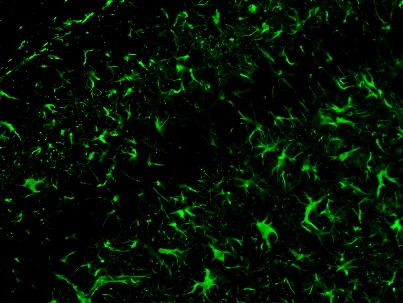

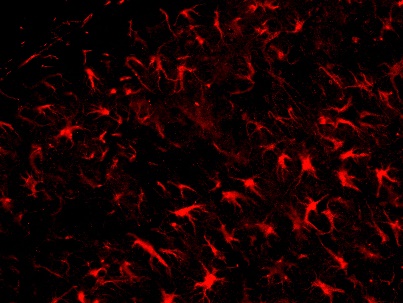


(B)


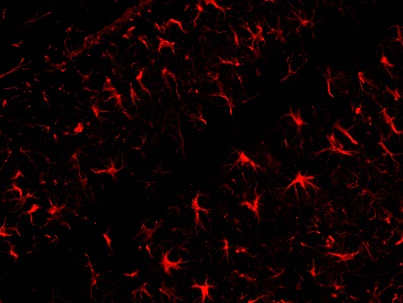

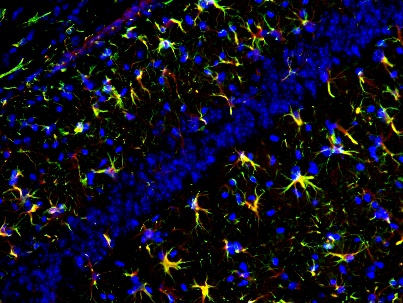

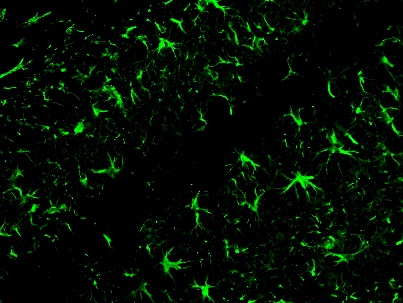


1. The original microscopy images of the SE+CMC group in Figure 3J
2. The original microscopy images of the SE+DMF group in Figure 3J

**Figure 4A**

(A)


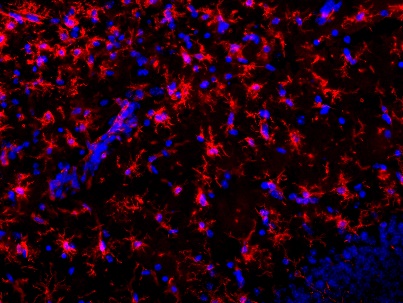

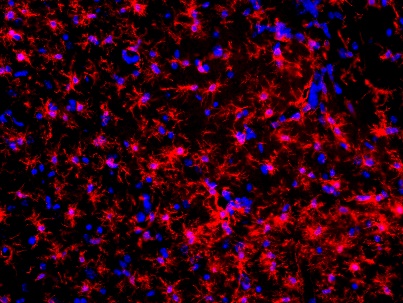

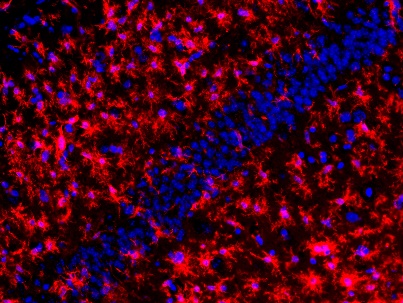


(B)


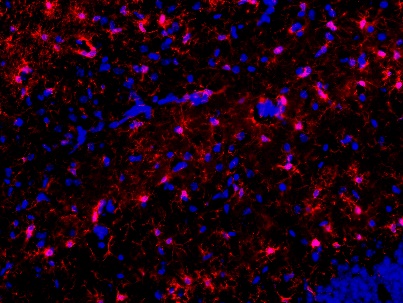

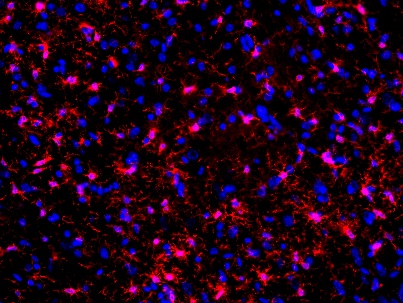

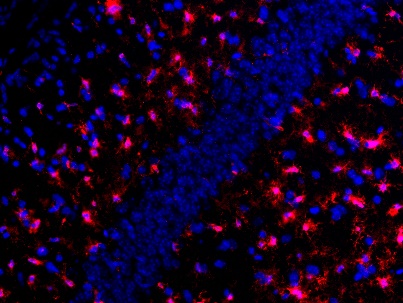


(A) The original microscopy images of the SE+CMC group in Figure 4A

(B) The original microscopy images of the SE+DMF group in Figure 4A

**Figure S1**

(A)


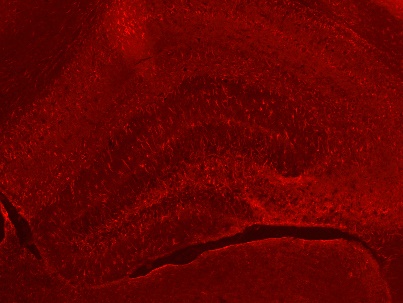

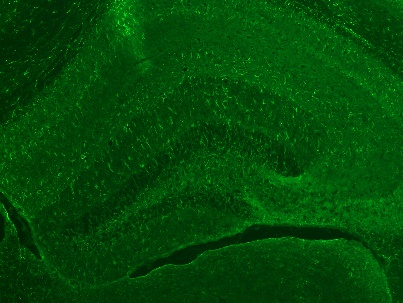

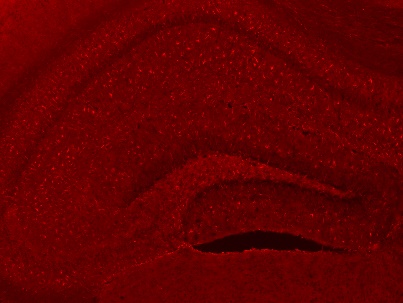

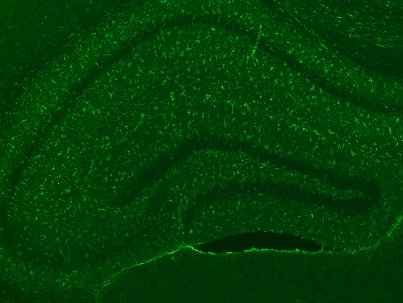


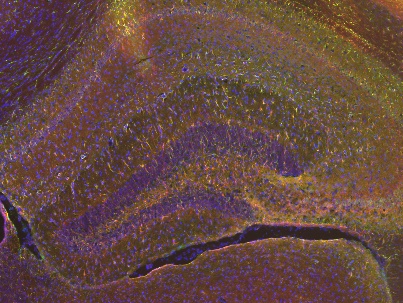

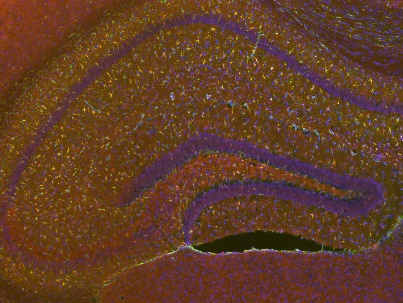


(B)


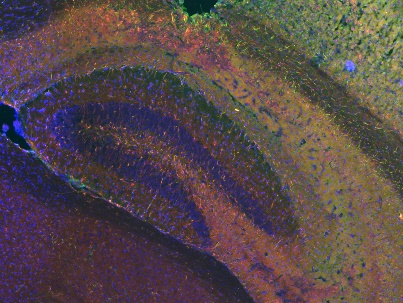

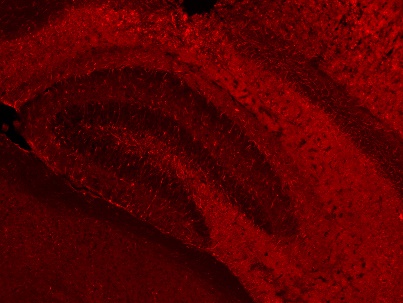

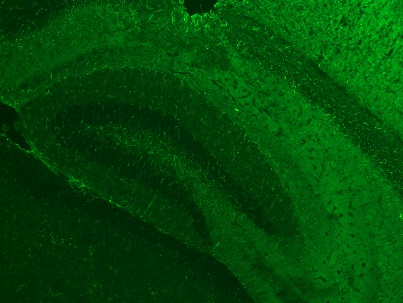

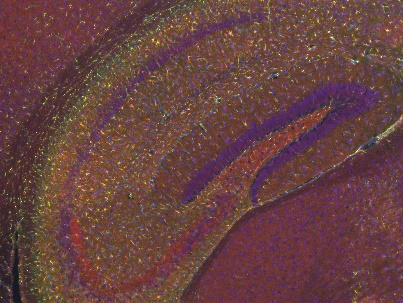

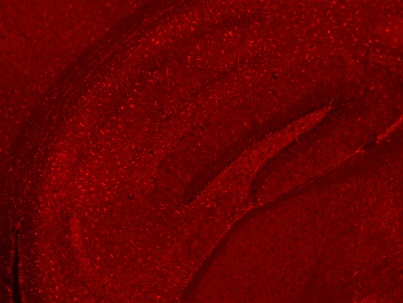

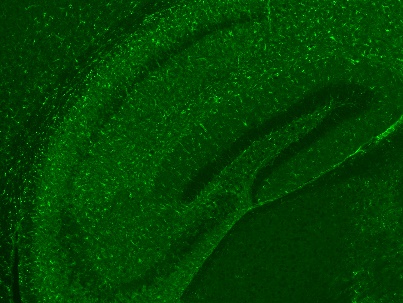


1. (B) The original microscopy images in Figure S1

**Figure S2**

（A）


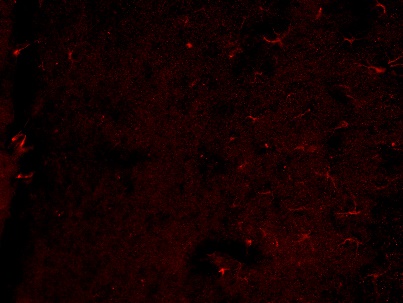

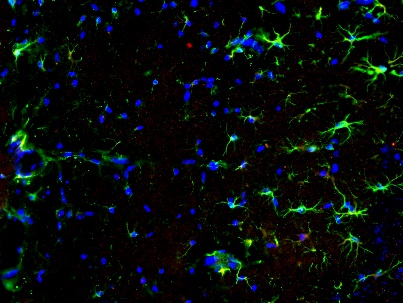

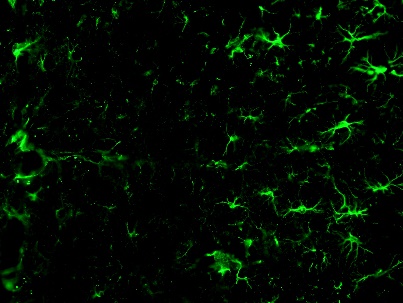

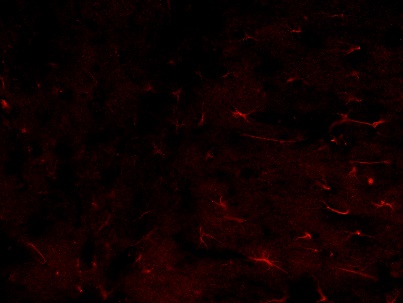

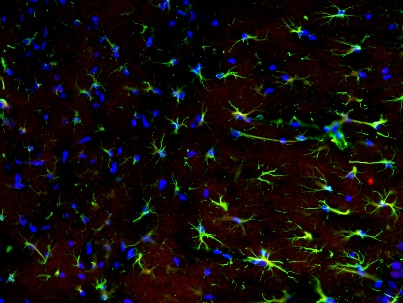

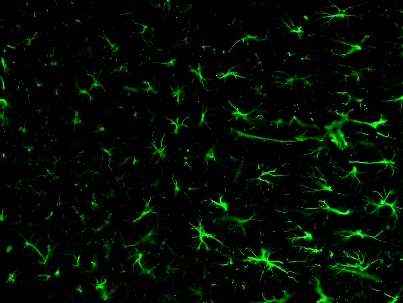


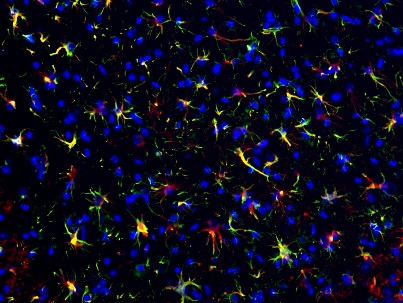

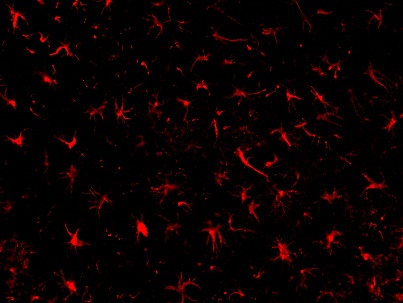

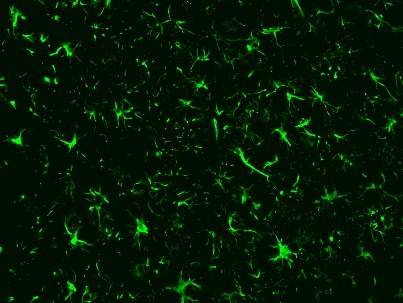


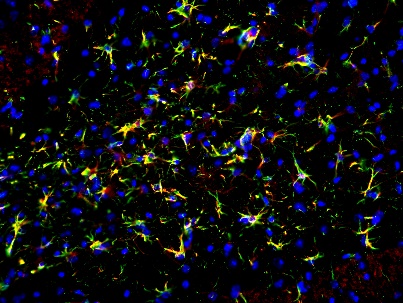

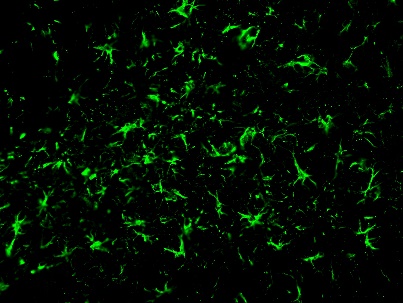

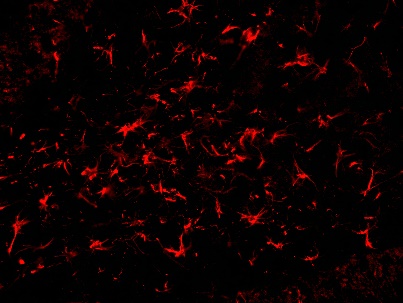


1. The original microscopy images of CA3 in Figure S2

**Figure S2**

(B)


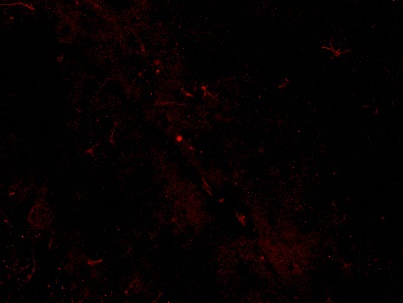

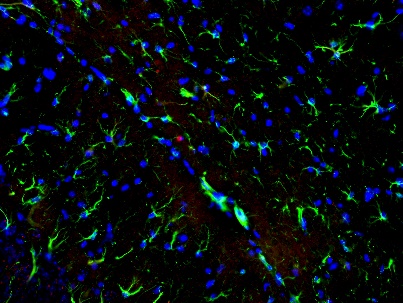

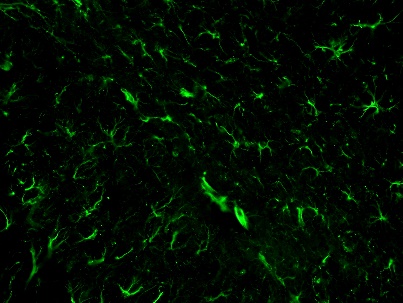


1. The original microscopy images of DG in Figure S2

**Figure S3**

(A)

(B)

(A) The original microscopy images of CA3 in Figure S3

(B) The original microscopy images of DG in Figure S3
